# Associations of the calcium-sensing receptor gene *CASR* rs7652589 SNP with nephrolithiasis and secondary hyperparathyroidism in haemodialysis patients

**DOI:** 10.1038/srep35188

**Published:** 2016-10-14

**Authors:** Alicja E. Grzegorzewska, Mateusz Paciorkowski, Adrianna Mostowska, Bartosz Frycz, Wojciech Warchoł, Ireneusz Stolarek, Marek Figlerowicz, Paweł P. Jagodziński

**Affiliations:** 1 Department of Nephrology, Transplantology and Internal Diseases, Poznan University of Medical Sciences, Poznań, Poland; 2 Department of Internal Diseases, Pleszew Medical Center, Pleszew, Poland; 3 Department of Biochemistry and Molecular Biology, Poznan University of Medical Sciences, Poznań, Poland; 4 Department of Biophysics, Poznan University of Medical Sciences, Poznań, Poland; 5 Institute of Bioorganic Chemistry, Polish Academy of Sciences, Poznań, Poland

## Abstract

Nephrolithiasis, secondary hyperparathyroidism (sHPT), and cardiovascular complications are associated with disturbances in Ca handling and contribute to morbidity/mortality during haemodialysis (HD). Calcimimetics, activators of the calcium-sensing receptor (CaSR), provide an effective means of reducing parathyroid hormone (PTH) secretion in sHPT. Polymorphism in CaSR gene (*CASR*) influences Ca-related parameters, however it was not shown in HD patients for *CASR* rs7652589. The minor allele at this polymorphism modifies the binding sites of transcription factors and CaSR expression. We hypothesized that *CASR* rs7652589 variants may also influence CaSR in end stage renal disease (ESRD). We aimed to determine the associations of rs7652589 with nephrolithiasis-related ESRD, Ca, P, ALP, PTH, response to treatment with cinacalcet, prevalence of coronary artery disease, and all-cause/cardiovascular mortality in HD patients (n = 1162). Healthy individuals (n = 918) were controls. This study shows that the A allele of rs7652589 is a risk allele for nephrolithiasis-related ESRD. The AA genotype is associated with more severe sHPT (higher Ca and PTH concentrations). The A allele is **as**sociated with reduced CaSR transcript level in peripheral blood mononuclear cells. According to computational analysis, potential binding sites for GLI3, AHR and TP53 are removed by the A allele, whereas binding sites for SOX18 and TP63 are created.

Calcium (Ca) stones contribute to nephrolithiasis-related end-stage renal disease (ESRD), which requires renal replacement therapy (RRT) in 26.7% of cases. Struvite stones (in humans, a mixture of struvite and carbonate-apatite^1^) account for a further 42.2% of cases^2^. Risk calcium-sensing receptor gene (*CASR*) genotypes in rs1042636, rs7652589, rs1501899, and rs6776158 were proposed as markers that could identify patients prone to developing Ca nephrolithiasis^3^,^4^. It was suggested that the appearance of the binding site for the octamer-binding transcription factor 1 (Oct-1) proximal to *CASR* promoters may downregulate calcium-sensing receptor (CaSR) expression in subjects with the minor allele in rs7652589 or rs1501899^5^. It was also demonstrated that the minor allele in rs6776158 may predispose to Ca stones by decreasing transcriptional activity of the *CASR* promoter 1 and CaSR expression in kidney tubules^4^.

Mineral bone disorders, including secondary hyperparathyroidism (sHPT), are closely associated with cardiovascular complications and are the main cause of morbidity and mortality in haemodialysis (HD) subjects^6^. CaSR expression is decreased in human uremic parathyroid glands to nearly 60% of normal expressionα^7^. Calcimimetics, allosteric activators of CaSR, provide an effective means of reducing parathyroid hormone (PTH) secretion in such patients^8^. Serum PTH concentrations and other features of sHPT^9^,^10^, as well as treatment with cinacalcet^11^,^12^, seem to be associated with *CASR* (OMIM +601199) polymorphisms. The *CASR* A986S (rs1801725) polymorphism was also associated with coronary artery disease (CAD), myocardial infarction (MI), all-cause mortality, and cardiovascular mortality in non-dialyzed subjects^13^.

CaSR in parathyroid cells is sensitive to ionized Ca concentration and regulates PTH secretion^14^. Blood ionized Ca is associated with polymorphisms in *CASR* A986S (rs1801725), R990G (rs1042636), and Q1011E (rs1801726)^15^,^16^. In 2000, Yano *et al*.^9^ demonstrated the association of *CASR* alleles (codon 990) with PTH secretion in HD patients. In 2001, Yamauchi *et al*.^17^ reported that *CASR* polymorphisms of G990R and intron 5 were closely associated with the magnitude of PTH secretion and/or PTH degradation as well as the clinical severity in primary HPT patients. In 2002, the *CASR* R990G polymorphism was shown to influence the response of the parathyroid glands to changes in extracellular ionized Ca in HD patients. The glands of patients with the GG genotype were more sensitive to extracellular changes in ionized Ca^10^.

Studies of the association between *CASR* and the response to treatment with the calcimimetic agent cinacalcet included *CASR* R990G. These studies showed that *CASR* R990G influences the response to calcimimetics in patients with sHPT with an odds ratio of 2.6^12^. Cinacalcet was also effective with a heterozygous mutation (R185Q, CGA > CAA) in exon 4 of *CASR*^18^. However, the *CASR* R990G polymorphism, which was investigated in small groups (n ≤ 20) of patients with primary HPT related to type 1 multiple endocrine neoplasia and in patients with sHPT, was not associated with the efficacy profile of cinacalcet^19^.

Vascular calcification is accelerated in the presence of elevated circulating Ca and phosphorus (P) concentrations^20^. Coronary artery calcification contributes to the development and progression of CAD^21^,^22^. Calcification is associated with the loss of functional CaSR in vascular smooth muscle cells, and this loss may be influenced by genetic factors (*CASR*, R185Q)^23^. Calcimimetics delayed the onset of calcification in vascular smooth muscle cells^23^.

In our study, the *CASR* promoter-near region single nucleotide polymorphism (SNP) rs7652589 was selected for genotyping. It has been shown that this variant and rs1501899 predispose individuals to idiopathic Ca nephrolithiasis^5^,^24^,^25^. Both of these SNPs were also associated with a specific phenotype in primary HPT patients characterized by an increased susceptibility to kidney stones and higher serum concentrations of ionized Ca and PTH^25^. We hypothesized that *CASR* rs7652589 variants may also influence CaSR in chronic kidney disease, particularly in ESRD. To the best of our knowledge, the *CASR* rs7652589 SNP has not been investigated in HD patients. The aim of the study was to determine the associations of the *CASR* rs7652589 SNP with nephrolithiasis-related ESRD, Ca, P, alkaline phosphatase (ALP), PTH, response to treatment with cinacalcet, prevalence of CAD, including MI, as well as all-cause and cardiovascular mortality in HD patients. Additionally, we aimed to investigate CaSR transcript level in peripheral blood mononuclear cells (PBMC) and to perform *in silico* prediction of transcription factor binding site (TFBS) overlapping *CASR* rs7652589.

## Results

### Patient characteristics

The demographic, clinical and laboratory data of the entire HD group as well as the demographic, clinical and laboratory data of subjects treated with cinacalcet are presented in Table 1. Among patients with nephrolithiasis-related ESRD (n = 108), there were 42 subjects with chronic infective tubulointerstitial nephritis (3.6% of all HD patients, 38.9% of nephrolithiasis-related ESRD subjects). Kidney stone composition was known in 11 patients. Calcium oxalate stones were diagnosed in all these cases. Primary gout or cystinuria were not diagnosed among the studied HD patients. In 10 out of 42 subjects who developed chronic infective tubulointerstitial nephritis in the course of renal stone disease, staghorn calculi were demonstrated after 1–3 decades since the detection of the first stone.

### Frequencies of *CASR* rs7652589 variants in HD patients and controls

A power analysis predicted sufficient sample power (>90%) to detect associations between the *CASR* rs7652589 SNP and ESRD requiring RRT with a genetic effect of 1.25 or greater in the dominant, recessive or log-additive modes of inheritance (Supplementary Table 1). There were no significant differences in the frequencies of *CASR* rs7652589 polymorphic variants between the entire group of HD subjects and healthy controls (Supplementary Table 2).

### Association of *CASR* rs7652589 SNP with HD patient data

Among HD patients, there were 959 (82.5%) subjects with sHPT (serum PTH > 150 pg/ml). Their genotype frequencies (GG 36%, AG 49%, AA 15%) did not differ from those of healthy subjects.

Comparisons of clinical and laboratory data of HD patients stratified by *CASR* rs7652589 genotypes suggested the possibility of associations (P < 0.05 without the Bonferroni correction) between *CASR* rs7652589 SNP and patient age at the start of RRT, nephrolithiasis-related ESRD, serum total Ca concentration, and prevalence of PTH results over 500 pg/ml (Supplementary Table 3).

Patients with the *CASR* rs7652589 AA genotype started RRT earlier than subjects with the GG genotype (Supplementary Table 3); however, patients who were aged < 40 years at the onset of RRT (n = 169) exhibited the GG genotype in 38% of cases, the AG genotype in 47% of cases, and the AA genotype in 15% of cases. These frequencies were not different from the respective frequencies (36%, 49%, and 15%) in patients starting RRT at age ≥ 40 years (n = 993).

Prevalence of nephrolithiasis-related ESRD significantly differed in patients stratified by *CASR* rs7652589 genotype (Supplementary Table 3). Compared to patients with the GG genotype, patients with the AA genotype (the additive model of inheritance) exhibited a higher frequency of nephrolithiasis-related ESRD, and the frequency of the A allele was higher in patients with nephrolithiasis-related ESRD than in other patients (Table 2). An association between the *CASR* rs7652589 SNP and nephrolithiasis remained significant after adjusting for gender and age at the onset of RRT (Supplementary Table 4). For the additive model of inheritance, the sample power was 99.8% with a genetic effect of 2.00 or greater, which was sufficient for the establishment of a significant association (Supplementary Table 5).

**Figure 1:**
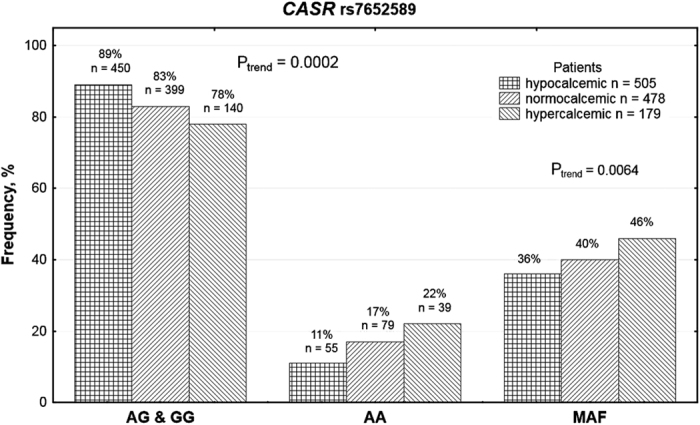
Association of *CASR* rs7652589 polymorphic variants with calcemic status of haemodialysis (HD) patients under conditions of taking Ca-containing phosphate binders . HD patients were stratified by categories of serum calcemic status: hypocalcemic (Ca < 8.80 mg/dl), normocalcemic (Ca 8.80–10.20 mg/dl), and hypercalcemic (Ca > 10.20 mg/dl). A significant trend for increasing frequencies of the A allele and the AA genotype was revealed among groups ordered as hypocalcemic, normocalcemic, and hypercalcemic.

Stratification of patients by *CASR* rs7652589 genotypes (Supplementary Table 3) suggested that the *CASR* rs7652589 A allele may be associated with higher serum concentrations of total Ca. Upon further analysis, HD patients were stratified by categories of serum calcemic status: hypocalcemic (Ca < 8.80 mg/dl), normocalcemic (Ca 8.80–10.20 mg/dl), and hypercalcemic (Ca > 10.20 mg/dl). If possible influences of Ca-containing drugs were not analyzed, the AA + AG patients showed higher serum Ca levels than the GG subjects (the dominant model of inheritance, P = 0.033, Supplementary Table 3). A significant trend for increasing frequencies of the A allele and the AA genotype was revealed among groups ordered as hypocalcemic, normocalcemic, and hypercalcemic (Fig. 1). Genetic comparisons between calcemic groups (Supplementary Tables 6–8) showed significant findings (P_trend_ = 0.001, P_genotype_ = 0.001) between hypercalcemic and hypocalcemic HD patients: hypercalcemic subjects exhibited a higher frequency of the A allele (OR 1.48, 95% CI 1.16–1.88, P = 0.002). The *CASR* rs7652589 genotype distribution yielded significance in the recessive (OR 2.28, 95% CI 1.45–3.58, P = 0.001) and the additive (OR 2.52, 95% CI 1.52–4.20, P = 0.001) models of inheritance (Supplementary Table 8). Taking into analysis the lowest Ca levels as possible less influenced by Ca-containing drug medication, we could observed significantly higher Ca concentrations in the AA patients compared with the GG (the additive model of inheritance, P = 0.000008) and the AA + AG (the recessive model of inheritance, P = 0.0000006) patients (Supplementary Table 3). In genetic comparisons, the most pronounced differences in distribution of *CASR* rs7652589 polymorphic variants were observed between hypocalcemic (n = 730) and normocalcemic (n = 396) subjects (P_trend_ <_ _0.0001, P_genotype _< 0.0001): normocalcemic subjects showed a higher frequency of the A allele (OR 1.52, 95% CI 1.28–1.82, P < 0.0001). The significance in distribution of *CASR* rs7652589 was revealed in the recessive (OR 2.69, 95% CI 1.92–3.76, P < 0.0001) and the additive (OR 2.82, 95% CI 1.94–4.10, P < 0.0001) models of inheritance. Small number of hypercalcemic patients among subjects, in whom possible treatment with phosphate binders was analyzed (n = 36), could be reason for a weak significance of genetic comparisons between hypercalcemic and hypocalcemic patients (P_trend_ = 0.169, P_genotype_ = 0.013).

In subjects stratified by *CASR* rs7652589 genotype, a higher prevalence of PTH results over 500 pg/ml was shown in patients harboring the AA genotype compared to patients bearing the G allele (Supplementary Table 3). Serum PTH levels over 500 pg/ml were associated with the allele A (Table 3).

There were no significant differences between groups stratified by *CASR* rs7652589 genotype in respect to serum phosphorus concentration (without or with possible influence of phosphate binders taken into the analysis), PTX rate or frequencies in cinacalcet administration, CAD or MI (Supplementary Table 3).

### Cytokine genes in nephrolithiasis-related ESRD

Among polymorphic variants of cytokine-associated genes tested in HD patients (Supplementary Table 9), borderline associations with nephrolithiasis-related ESRD were shown for the chemokine (C-C motif) ligand 2 gene (*CCL2* -2518 A > G) rs1024611 (P_*trend*_ = 0.059) and the interleukin (IL) 18 gene (*IL18*) rs360719 (P_*trend*_ = 0.085). The interaction analysis revealed a significant epistatic interaction between *CASR* rs7652589 and *CCL2* rs1024611 polymorphisms (OR for interaction 0.565; Chi-square statistic, 1df, 5.19; P = 0.023) (Supplementary Table 10).

### Vitamin D signaling pathway genes in nephrolithiasis-related ESRD

No significant differences in frequency distributions of polymorphic variants in vitamin D signaling pathway genes between HD patients with and without nephrolithiasis-related ESRD were observed (Supplementary Table 11). Associations between vitamin D signaling pathway genes and nephrolithiasis-related ESRD as well as interactions with *CASR* rs7652589 were also not observed (Supplementary Table 12).

### Genotypes as independent determinants of specific phenotypes

Gender, age, serum concentrations of Ca and PTH, the G allele of *CCL2* -2518 A > G (rs1024611), and the A allele of *CASR* rs7652589 were entered into the multivariate stepwise linear regression model as possible determinants of nephrolithiasis-related ESRD. Among these factors, only the A allele of *CASR* rs7652589 was an independent determinant of nephrolithiasis-related ESRD (B 0.09, 95% CI 0.01–0.17, P = 0.020).

Gender, RRT vintage, serum PTH level, serum P concentration, serum ALP activity, and the A allele of *CASR* rs7652589 were chosen as possible determinants of serum Ca concentration exceeding 10.2 mg/dl (the upper normal range) under conditions of taking Ca-containing phosphate binders. High serum Ca levels were determined in HD patients by serum concentrations of PTH (B 0.16, 95% CI 0.07–0.24, P = 0.0004) and P (B 0.11, 95% CI 0.04–0.19, P = 0.002), but not by the A allele of *CASR* rs7652589. A replacement of the A allele in this model by the AA genotype of *CASR* rs7652589 did not change the significance of the determinants. When serum Ca as a continuous variable was taken to the analysis, there were also no association of the *CASR* rs7652589 A allele or AA genotype with Ca level. The same abovementioned 6 parameters were applied as possible determinants of serum Ca concentration under conditions diminishing possible influences of Ca-containing drugs. Significant independent positive determinants of serum Ca remained female gender (B 0.08, 95% CI 0.02–0.15, P = 0.017) and the AA genotype of *CASR* rs7652589 (B 0.14, 95% CI 0.07–0.21, P = 0.00008), but not the A allele if used instead of the AA genotype.

Gender, RRT vintage, serum P concentration, serum ALP activity, and the A allele of *CASR* rs7652589 were chosen as possible determinants of serum PTH levels exceeding 500 pg/ml. These PTH levels were determined in HD patients by RRT vintage (B 0.21, 95% CI 0.14–0.27, P < 0.000001), serum P concentration (B 0.18, 95% CI 0.11–0.24, P < 0.000001), and serum ALP activity (B 0.25, 95% CI 0.17–0.32, P < 0.000001), but not by the A allele of *CASR* rs7652589. However, if the A allele was replaced in this model by the AA genotype of *CASR* rs7652589, the AA genotype was significantly related to serum PTH levels exceeding 500 pg/ml (B 0.09, 95% CI 0.02–0.015, P < 0.008). The remaining determinants maintained their significance.

### Response to treatment with cinacalcet

*CASR* genotypes of HD patients treated with cinacalcet were disclosed after collection of all data related to administration of this agent.

Initial PTH concentrations and maximum doses of cinacalcet were similar in the genotype-stratified groups. There was no significant difference in prevalence of responders to cinacalcet with respect to the *CASR* rs7652589 polymorphism (Supplementary Table 13). Among the collected clinical/laboratory variables, significant determinants of sensitivity to treatment with cinacalcet were lower ALP activity (B −0.19, 95% CI −0.35–0.03, P = 0.023) and lower maximal doses of cinacalcet used for treatment (B −0.19, 95% CI −0.36 – −0.03, P = 0.019).

### Association of the *CASR* rs7652589 SNP with clinical outcomes of HD patients

A total of 449 HD patients died, and 270 underwent renal transplantation between January 2nd, 2009 and April 20th, 2015.

**Figure 2:**
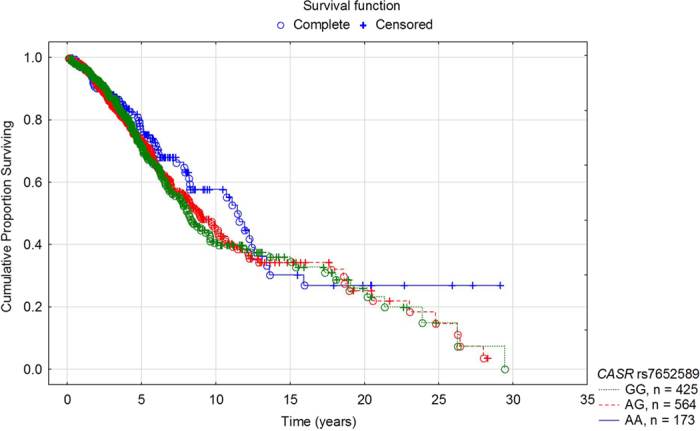
The Kaplan-Meier survival analysis of the studied haemodialysis patients since the initiation of renal replacement therapy stratified by *CASR* rs7652589 genotypes. Patients’ survival was not dependent on the tested genotypes (the log-rank test P = 0.449).

Using the Kaplan-Meier curve estimate in the entire cohort of HD patients, *CASR* rs7652589 genotypes did not have an effect on all-cause mortality (Fig. 2). Significance was also not revealed when the analysis was performed using models of inheritance (Supplementary Figures 1–3). Neither cardiac mortality nor cardiovascular mortality rates were associated with the *CASR* rs7652589 SNP (data not shown, P > 0.05).

### Effect of *CASR* rs7652589 G>A polymorphism on CaSR transcript levels in PBMC from HD patients

**Figure 3:**
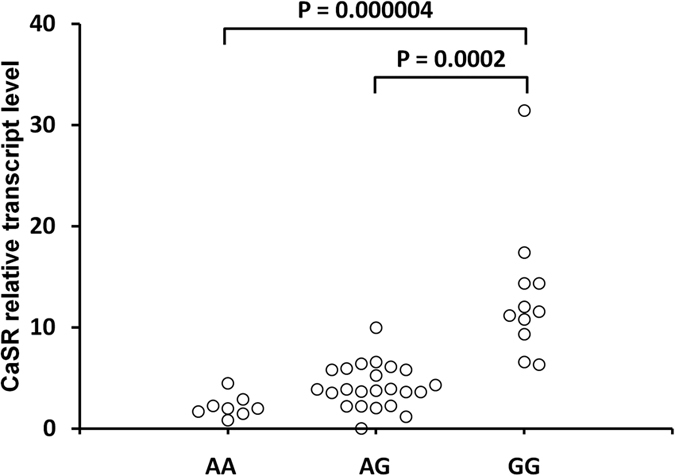
Effect of the *CASR* rs7652589 G>A polymorphism on CaSR transcript levels in PBMC obtained from HD patients. The PBMC from whole venous blood were isolated by Ficoll-Hypaque centrifugation (density, 1.077 g/cm^3^) followed by total RNA isolation. Quantitative analyses of CaSR transcript levels were performed by RT and Q-PCR SYBR Green I system. The quantity of CaSR transcript in each sample was standardized by the geometric mean of reference PBGD and B2M cDNA levels.

We found a reduced CaSR transcript level in the PBMC from carriers of the *CASR* rs7652589 A allele as compared with carriers of the *CASR* GG genotype. The median of CaSR transcript level in PBMC was 2.00 (range 0.86–4.49) for the AA genotype, 3.90 (range 0.03–9.98) for the AG genotype, and 11.59 (range 6.34–31.44) for the GG genotype (Fig. 3).

### *In silico* TFBS prediction

ENCODE TFBS ChiP-seq dataset reports 5 transcription factors (CTCF, ELK4, KAP1, POLR2A, and TCF7L2) tested in the HEK293 cells with none having DNA-binding sites overlapping rs7652589 position. Furthermore, none of the 161 ChiP-seq analyzed transcription factors in all ENCODE cell lines contains a DNA-binding site overlapping rs7652589 (Table 4).

The computational analysis predicted removal of binding sites for the transcriptional activator GLI3, aryl hydrocarbon receptor (AHR), and cellular tumor antigen p53 (TP53) at the applied statistical significance cut off in the presence of the A allele. Binding sites for the transcription factor SOX18 and tumor protein 63 (TP63) however, were added in the presence of the A allele. Binding site for the PO2F1 motif, of which Oct-1 is a member, was not found in the presence of the A allele at the applied statistical significance cut off (Table 4).

## Discussion

In this study, we have shown that an epistatic interaction between the *CASR* rs7652589 SNP (risk allele A) and *CCL2* rs1024611 (risk allele G) is associated with nephrolithiasis-related ESRD requiring regular dialysis treatment. The A allele of *CASR* rs7652589 was also a significant determinant of nephrolithiasis-related ESRD among other possible determinants such as gender, age, serum concentrations of Ca and PTH, and the G allele of *CCL2* rs1024611. The AA genotype of *CASR* rs7652589 SNP appears to be independently related to higher concentrations of serum total Ca and higher frequency of serum PTH concentrations exceeding 500 pg/ml, but not to serum P concentration and serum total ALP activity. Prevalence of CAD, including MI, response to treatment with cinacalcet hydrochloride, as well as all-cause and cardiovascular mortality since the onset of RRT were not associated with the *CASR* rs7652589 SNP. These results were not influenced by the possible association of the *CASR* rs7652589 polymorphism with end-stage renal failure as a general phenotype.

Nephrolithiasis was reported in 6.6% of the elderly Polish population^26^, and severe nephrolithiasis leading to infective tubulointerstitial nephritis was shown in 2.4% of dialysis patients in Poland^27^. In as many as 9.3% of the studied HD patients, a long-term presence of nephrolithiasis and recurrent formation of calculi, as well as related complications, became a cause of progressive chronic kidney disease (chronic infective tubulointerstitial nephritis, obstructive nephropathy, and pyonephrosis with subsequent nephrectomy) leading to RRT. Prevalence of nephrolithiasis related ESRD in the studied group was higher than that previously described (up to 4.7% in the period 1989–1991 in France)^2^. This difference may be due to inclusion in our nephrolithiasis related ESRD group not only patients with chronic or recurrent obstruction of the urinary tract (obstructive nephropathy) but also subjects in whom chronic infective tubulointerstitial nephritis was diagnosed as related to stone formation without obstruction of the urinary tract as the main clinical problem.

HD patients having nephrolithiasis as a cause of ESRD differed in the *CASR* rs7652589 SNP compared with other HD patients. The A allele of *CASR* rs7652589 was recognized in our study as a risk allele for severe nephrolithiasis, among other possible factors, such as gender, age, serum concentrations of Ca and PTH, and the G allele of *CCL2* rs1024611.

It should be noted that *CCL2* rs1024611 and *IL18* rs360719 yielded borderline associations with nephrolithiasis-related ESRD simultaneously with a significant association with the *CASR* rs7652589 SNP.

The G allele of *CCL2* rs1024611, which is known to be a risk allele for inflammatory infective and non-infective diseases^28^,^29^, could simultaneously participate in progressing chronic kidney disease in nephrolithiasis patients as indicated by the interaction of *CCL2* rs1024611 with *CASR* rs7652589 in the studied HD subjects. Moreover, *CCL2* expression was found to be induced by calcium oxalate monohydrate in renal epithelial cells^30^. Suppression of *CCL2* mRNA expression and downregulation of the expression of *CCL2*-related proteins including transforming growth factor beta-1 by resveratrol in oxalate-treated human renal epithelial cells significantly reduced the number of urine calcium oxalate crystals^31^. Patients harboring the G allele of *CCL2* rs1024611, which was also associated with increased CCL-2 production^32^ in HD patients^33^, could be particularly prone to increased *CCL2* expression contributing to interstitial inflammation and fibrosis^34^ and, therefore, to the initiation of chronic kidney disease and the progression of this disease to ESRD. A significant epistatic interaction between *CASR* rs7652589 and *CCL2* rs1024611 polymorphisms is in accordance with this concept.

*IL18* rs360719 did not exhibit an epistatic interaction with *CASR* rs1501899. This SNP was associated with chronic infective tubulointerstitial nephritis as a cause of ESRD in our previous study^35^. HD patients with nephrolithiasis-related ESRD showed chronic infective tubulointerstitial nephritis in only 38.9% of subjects. This fact can explain the borderline association of *IL18* rs360719.

The *CASR* rs7652589 SNP, together with the *CASR* rs1501899 SNP, was involved in kidney stone production in primary HPT patients; however, the allele frequency at the rs7652589 SNP was similar in primary HPT patients and controls^25^. HD patients generally exhibited sHPT–82.5% in the current study. The first calculi were identified in our patients long before the development of sHPT. Patients with sHPT did not differ from healthy subjects in their distribution of the *CASR* rs7652589 SNP.

HD patients harboring the *CASR* rs7652589 AA genotype more frequently exhibited serum PTH levels exceeding 500 pg/ml; therefore, they were prone to more severe sHPT than the remaining subjects. Moreover, they presented with higher serum levels of total calcium. If possible influences of Ca-containing drugs were at least partially eliminated from the analysis, the AA genotype appeared significant independent determinant of higher Ca levels. Similarly, subjects with primary HPT having the AA haplotypes of the rs7652589 and rs1501899 SNPs demonstrated higher ionized Ca concentrations than patients harboring the other *CASR* haplotypes^25^. Hypercalcemic sHPT usually indicates tertiary HPT. HD patients bearing the *CASR* rs7652589 AA genotype seem to be more prone to this severe form of sHPT. Generally, our results have shown that associations of the *CASR* rs7652589 SNP with serum concentrations of Ca and PTH that were observed in primary HPT patients^25^ are also present in ESRD patients regularly treated with HD, in whom sHPT is a common finding.

No association of the *CASR* rs7652589 SNP with serum activity of total ALP was found in the tested HD patients. Serum total ALP activity reflects alkaline phosphohydrolases of tissue-specific origins such as bone, liver, and intestine^36^. A lack of association, if any exists, may be due to total ALP non-specificity.

Recently, Babinsky *et al*.^37^ did not reveal differences in *CASR* rs7652589 genotype distribution in kidney transplant recipients showing low or high levels of aortic calcification and coronary artery calcification. The *CASR* rs7652589 SNP was not associated with cardiovascular outcomes or mortality in this cohort^37^. Our study did not indicate any associations between this SNP and prevalence of CAD, including myocardial infarction, as well as all-cause and cardiovascular mortality rates in HD patients.

Calcimimetics increase CaSR expression^38^ and are used to reduce PTH secretion by parathyroid cells in sHPT patients^8^. A decrease in serum PTH concentration in response to administration of cinacalcet varies between HD patients but, according to our results, these differences in responsiveness are not dependent on the *CASR* rs7652589 SNP. However, our study group was too small to allow us to draw definitive conclusions.

It was demonstrated that Ca nephrolithiasis is associated with a decreased CaSR expression in kidney tubules^4^, and uremic parathyroid glands show a decreased CaSR expression^6^. We observed significantly reduced CaSR transcript levels in the PBMC from carriers of the *CASR* rs7652589 A allele as compared to carriers of the *CASR* GG genotype. Lower expression of CaSR was associated with higher levels of Ca and PTH in the *CASR* rs7652589 AA genotype carriers in our study. Although we were able to examine a CaSR expression only in PBMC, an association of reduced transcript levels with A allele indicates that *CASR* rs7652589 may be involved in Ca-related disturbances occurring due to CaSR downregulation.

The tested *CASR* rs7652589 variant is located 13 kbp upstream from the TATA box of promoter 1 on human chromosome 3. Our *in silico* analysis confirmed that the minor allele at this polymorphism is able to modify the binding sites of specific transcription factors and, consequently, *CASR* expression. We have shown that potential binding sites for GLI3, AHR and TP53 are removed by the A allele, whereas binding sites for SOX18 and TP63 are added. We performed an analysis of the PO2F1 motif, of which Oct-1 is a member. In contrast to Vezzoli *et al*.^5^, our analysis does not support appearance of Oct-1 in presence of the A allele. This finding likely stems from the software differences between studies, as we find, that p-value alone of the PO2F1 motif is statistically highly significant. However applied in our work, false discovery rate filter marked PO2F1 motif above cut-off criteria. Therefore, our study suggests that Oct-1 may not be a down-regulator of CaSR expression in the A allele harbouring subjects as it was postulated by Vezzoli *et al*.^5^. It is still unsolved how binding sites for the transcription factor SOX18 and tumor protein 63, that are added in the presence of the A allele, may influence a CaSR expression.

### Limitations of the study

Associations of *CASR* rs7652589 with selected phenotypes were analyzed under conditions of conventional treatment targeting sHPT. Ca-containing drugs were administered the most commonly, serving as phosphate binders and Ca supplements. These drugs were usually started still prior to HD therapy, and their discontinuation was not possible due to ethical reasons. However, evaluations of patient compliance reveal poor adherence with drugs prescriptions in HD subjects: half were noncompliant with medication taking^39^. Poor compliance could be a reason of decreases in serum Ca levels and increases in P concentrations during the prescribed treatment.

It has to be pointed out that we have not applied the Bonferroni corrected P value in analyses of the *CASR* association with 8 main phenotypes tested in this study (nephrolithiasis-related ESRD, Ca, P, ALP, PTH, response to treatment with cinacalcet, prevalence of coronary artery disease, and survival probability). Associations of *CASR*, presented in our paper as significant, were obtained at P value below 0.05 but in some analyses over 0.006. However, a significance of polymorphic variant as a determinant of a specific phenotype was verified among other clinically important variables, and only these *CASR* variants which remained significant were considered as significantly associated. Although the Bonferroni correction is still widely used, there is a criticism to this method^40^ and not all authors are willing to use it in genetic studies^41^,^42^,^43^, especially when the established associations are investigated in more detail or in other uniform cohort like in our study. When several phenotypes is simultaneously tested, the use of Bonferroni correction may exclude some important associations.

## Patients and Methods

### Patients

ESRD patients whose current method of RRT was intermittent HD were enrolled in the study. Patient enrollment began in January, 2009 and ended in May, 2014. At enrollment, patients were dialyzed in 21 dialysis centers in the Greater Poland region of Poland. The patient group included 1162 individuals. Nephrolithiasis-related ESRD was diagnosed if calculus formation caused obstructive nephropathy and/or chronic infective tubulointerstitial nephritis. CAD was diagnosed based on a medical history, electrocardiograms, exercise stress test, and, in some cases, coronary angiography or computed tomography. MI was diagnosed based on a medical history and evidence showing characteristic electrocardiographic abnormalities and elevated levels of cardiac markers of cardiomyocyte damage.

In all HD patients, therapeutic efforts were aimed at reaching the normal serum levels of Ca and P and maintaining serum intact PTH levels at a range of approximately 2 to 9 times the upper normal limit for the assay in accordance with the Kidney Disease: Improving Global Outcomes (KDIGO) Work Group clinical practice guidelines^44^. Patients with serum PTH levels equal to or exceeding 500 pg/ml could receive treatment with cinacalcet hydrochloride (reimbursed by the National Health Fund - NHF). HD patients with sHPT who failed to respond to pharmacological treatment presented with PTH levels exceeding 1000 pg/ml in repeated evaluations and were tested for parathyroid gland adenoma on 99mTc-MIBI scintigraphy. Parathyroidectomy (PTX) was performed if possible (no clinical contraindications, written informed consent obtained).

Treatment with cinacalcet (Mimpara, Amgen Europe, Breda, Netherlands) at a daily dose of 30 mg was started in 162 patients with PTH exceeding 500 pg/ml. Patients who showed serum PTH of 1000 pg/ml or greater before the planned treatment with cinacalcet underwent 99mTc-MIBI scintigraphy. Only those without scintigraphic suspicion of parathyroid gland adenoma qualified for this treatment. Serum PTH was checked in the treated group every 4 weeks, and cinacalcet doses were increased up to 180 mg per day, if well tolerated, until the PTH concentration decreased at least by 40% of the initial level, which was considered as a satisfactory response to treatment. Patients who did not exhibit sufficiently decreased serum PTH were referred to as non-responders to cinacalcet.

The principal dietary and pharmacological treatment of all patients was based on a standard of care according to the physician. Guidelines for this treatment based on recommendations of the Polish Society of Nephrology and the principal country consultants in nephrology. Nephrologists had been prescribing calcium carbonate or calcium acetate in 1031 cases due to hyperphosphataemia without hypercalcaemia or due to hypocalcaemia. Sevelamer hydrochloride was used in 24 hypercalcaemic/hyperphosphatemic patients. Frequency of patients receiving phosphate binders did not differ in groups stratified by *CASR* polymorphic variants (Supplementary Table 3). Among vitamin D supplements, alfacalcidol (Alfadiol, GlaxoSmithKline Pharmaceuticals SA, Poland) was the most frequently used. None of patients was administered intravenous vitamin D analogues. Patients were using erythropoietin stimulating agents, iron and water-soluble vitamins. Co-morbid conditions were treated as needed.

Blood samples for Ca, P, ALP, and PTH were routinely taken from all HD patients at the onset of RRT. Periodical measurements were conducted every 1–3 months for Ca and P and every 6–12 months for ALP and PTH. Differences in the frequency of measurements were dependent on the intrinsic regulations of the respective dialysis centers.

A majority of the HD patients enrolled in the current study were previously genotyped for T helper (Th) cell cytokine-associated genes, including *CCL2* -2518 A>G rs1024611 that is also referred to as the monocyte chemotactic protein 1 gene (*MCP1*). Patients were also genotyped for vitamin D signaling pathway genes^35^,^45^,^46^. The frequency distributions of polymorphic variants of these genes and their associations with the tested phenotypes, as well as their epistatic interactions with *CASR* rs7652589, were analyzed in HD patients in the ongoing study.

### Controls

Healthy individuals (blood donors and healthy volunteers unrelated to the patients and to each other) from the same region of Poland served as controls for the frequency distribution of the *CASR* rs7652589 SNP. The control group included 918 subjects.

All study subjects were Caucasians of Polish origin.

### Patient Data

Demographic and clinical parameters including date of birth, gender, chronic kidney disease causing ESRD and requiring RRT, date of the start of RRT, presence of CAD, a history of MI and PTX were recorded by a review of the medical charts and by personal communications with the nephrologists responsible for the treatment of patients in the dialysis centers. The patient data were evaluated every year from the initiation to the end of the enrollment period with respect to the diagnosis of CAD and the history of MI and PTX.

Laboratory parameters included serum concentrations of total Ca, P, and intact PTH, as well as serum activity of total ALP; all serum concentrations were determined using routine laboratory methods by laboratories under agreement with the dialysis centers. The laboratory data were evaluated in our database every year: the last four results obtained while the patient was in stable condition were collected as long as their number was not limited by a short HD vintage. For analysis, each laboratory parameter was represented as a mean value of up to 4 results obtained during the last period of HD vintage. In patients who underwent PTX or were treated with cinacalcet, laboratory data obtained before those treatments were included in the analysis. Analyses of the *CASR* association with serum Ca were also performed using the lowest Ca levels among up to 4 last collected values, if patients were receiving Ca-containing drugs. The *CASR* association with circulating P were also explored using the highest P levels among up to 4 last collected values, if patients were taking phosphate-binding drugs.

Additionally, plasma 25(OH)D levels were determined in 217 blindly selected HD patients. Blood samples for 25(OH)D were taken in the winter to avoid differences in sunlight exposure between patients who sunbathed and those who did not. Plasma 25(OH)D concentrations were measured in HD patients who had not been treated with vitamin D or had stopped vitamin D treatment at least 3 weeks earlier.

In patients treated with cinacalcet, serum PTH concentrations preceding the initiation of cinacalcet administration and those measured 1–2 months after implementation of the maximum cinacalcet dose were collected; however, serum PTH concentrations were collected no longer than 12 months from the beginning of treatment.

For the survival analysis, the dates of kidney transplantation, movement to a non-collaborating dialysis center, or death were recorded. The causes of death were recorded and categorized as cardiac (reported as myocardial infarction, sudden cardiac death, severe arrhythmias, cardiomyopathies, or cardiac failure), vascular (reported as cerebrovascular events, cerebral stroke, or generalized atherosclerosis), or other. The last evaluation of patient data was performed between March 27th and April 20th, 2015.

### Genotyping the *CASR* rs7652589 polymorphism

The *CASR* rs7652589 SNP was genotyped using leukocyte DNA obtained from 1162 HD patients and 918 controls. High-resolution melting curve analysis (HRM) was performed using a LightCycler 480 system (Roche Diagnostics, Mannheim, Germany). The 178 bp PCR product was amplified using the following primers: F: 5′-ACTGCCCTCATCATTCCTTC-3′ and R: 5′-ATCATCCTCCCTGCAAGAAC-3′. The annealing temperature was 61 °C. Amplified DNA fragments were subjected to HRM with temperatures ranging from 77 °C to 92 °C in 0.1 °C increments. Quality control was ensured by running 10% of the samples in duplicate.

### Real-time quantitative PCR (Q-PCR) analysis of CaSR transcript levels in PBMC

Blood samples (5 ml each) from 42 HD patients was collected into tubes containing EDTA. The study group included 8 individuals with AA, 23 with AG, and 11 with GG genotype. PBMC were separated by Ficoll-Hypaque centrifugation (density, 1.077 g/cm^3^) followed by total RNA isolation. Total RNA was isolated according to the method of Chomczyński and Sacchi^47^. RNA samples were treated with DNase I, quantified and reverse-transcribed (RT) into complimentary DNA (cDNA). Quantitative analysis of CaSR cDNA was performed by Light Cycler^®^480 II Real-Time PCR System (Roche Diagnostics GmbH, Mannheim, Germany), using SYBR Green I as detection dye. CaSR cDNA was quantified using the relative quantification method with a calibrator. The calibrator was prepared as a cDNA mix from all cDNA samples and consecutive dilutions were used to create a standard curve. For amplification, 1 μl of cDNA solution was added to 9 μl of LightCycler 480 SYBR Green I Master Mix (Roche Diagnostics GmbH, Mannheim, Germany) and primers. The quantity of CaSR transcript in each sample was standardized by the geometric mean of porphobilinogen deaminase (PBGD) and beta-2-microglobulin (B2M) transcript levels. The PCR amplification efficiency for target and reference cDNA was determined by different standard curves created by consecutive dilutions of the cDNA template mixture. The CaSR cDNA 96 bp amplicon was amplified using the primer pair: (forward 5′ ATGCCAAGAAGGGAGAAAGAC 3′) and (reverse 5′ GGAGGACAGACAGCACAAAG 3′). The PBGD 160 bp and B2M cDNA 106 bp amplicons was amplified using the primer pair: (forward 5′ GCC AAG GAC CAG GAC ATC 3′), (reverse 5′ TCA GGT ACA GTT GCC CAT C 3′) and (forward 5′ CACCCCCACTGAAAAAGATG 3′), (reverse 5′ CCTCCATGATGCTGCTTACA 3′), respectively. The CaSR mRNA levels were expressed as multiples of these cDNA concentrations in the calibrator.

### Statistical analysis

The results are presented as percentages for categorical variables or as the median and range (minimum - maximum) for continuous variables because the majority of continuous variables were non-normally distributed as tested by the Shapiro–Wilk test.

Clinical and laboratory data were compared in HD subjects stratified by *CASR* rs7652589 genotype. The results were analyzed using three inheritance models (dominant, recessive and additive). Continuous variables were compared using the Mann-Whitney test. For comparison of categorical variables, Chi-square test with Yates correction was used. Parameters for which this preliminary analysis suggested an association with *CASR* rs7652589 in at least one model of inheritance (P < 0.05 without the Bonferroni correction) were chosen for detailed statistical analysis as described below.

Statistical analysis of CaSR transcript level comparison between the AA vs GG and AG vs GG genotype carriers was evaluated by the *Kruskal***-***Wallis test*.

In this study, the power for detection of genetic associations was determined using Quanto v.1.2.4 software^48^. Departure from the Hardy-Weinberg equilibrium was determined by Chi-squared analysis. Polymorphisms were tested for trends in association using the Chi-square test if two groups were compared or the Cochran-Armitage test if three groups were compared (P_trend_). Genotype distributions were compared between the tested groups using the standard Chi-square test (P_genotype_). Odds ratios (OR) and 95% confidence intervals (CIs) for OR were calculated to assess the strength of the association with categorical variables. Chi-square test with Yates correction was used for statistical evaluation of OR. Additionally, gender and age at the onset of RRT (<40 years vs. ≥ 40 years) were adjusted for in analyses conducted using Cochran-Mantel-Haenszel statistics. All probabilities were two-tailed, and a Bonferroni corrected *P* value lower than 0.017 (1 SNP, 3 models, 1 phenotype) was considered significant.

Multiple regression analysis was performed to determine whether genotypes that were associated with specific phenotypes were also independent determinants of those phenotypes among other variables. The multivariable models included clinical characteristics of HD patients, laboratory parameters related to Ca and sHPT, and a *CASR* polymorphic variant tested for association. The chosen variables were entered into a stepwise multivariate linear regression model, and an assessment of confounding variables was performed. The results of the multivariate analysis are presented as the regression coefficient (B) values ± 95% CI.

Survival probability since the onset of RRT was analyzed with respect to *CASR* genotypes. This analysis was also performed using dominant, recessive and additive models of inheritance. The Kaplan-Meier method with the log-rank test was used to estimate significant differences in the cumulative proportion of survival curves characterizing the genotype groups in each model of inheritance.

The previously mentioned statistical analyses were performed using Graph-Pad InStat 3.10, 32 bit for Windows, created July 9, 2009 (GraphPad Software, Inc., San Diego, California, United States), CytelStudio version 10.0, created January 16, 2013 (CytelStudio Software Corporation, Cambridge, Massachusetts, United States), and Statistica version 12, 2014 (Stat Soft, Inc., Tulsa, Oklahoma, United States).

Epistatic interactions between *CASR* rs7652589 and Th cell cytokine-associated genes and vitamin D signaling pathway genes were analyzed in patients with and without nephrolithiasis-related ESRD using the logistic regression and epistasis option in the PLINK software (http://pngu.mgh.harvard.edu/purcell/plink/).

Values of P < 0.05 were considered significant in the epistatic interaction, multiple regression and survival probability analyses.

### *In silico* TFBS prediction

Postulated rs7652589 (G>A) regulatory impact on *CASR* expression through transcription factor binding sites motifs disruption^5^ was assessed with an analysis of the experimental ENCODE TFBS ChIP-seq data and *in silico* prediction of DNA-binding sites collected in HOCOMOCO version 9^49^ and JASPAR CORE version 5.0 ALPHA 2016^50^ databases with FIMO software version 4.11.1^51^. FIMO scans DNA sequences utilizing position-specific frequency matrices of the given motifs and reports the output as the P value computed from log-likelihood ratio score for each database motif and applies false discovery rate analysis to estimate q values.

Since ENCODE TFBS ChIp-seq experiments contain data for only 5 out of the total 161 TFs for the relevant HEK293 cell line, we bona fide expanded our DNA-binding sites search in rs7652589 containing region to the all ChIP-seq’ed cell lines.

We carried out the computational analysis on the GenBank DNA sequences (contig NT-005612.16) adjacent to rs7652589 positions. Two FASTA sequences, each with one of the rs7652589 alleles, were used as an input for the FIMO. To minimize both false positive and false negative rates, the background file was calculated directly from used input sequences with MEME-suit script^52^.

Results were analyzed via a self-developed Python script to find predicted transcription factor binding sites ranges containing rs7652589. A P value < 0.0005 and q value < 0.05 were selected as the cut-off values for reliable predictions. Motifs matching to both strands were also analyzed whether or not they form perfect reverse complements, therefore making the match more probable. We obtained information about identified transcription factors from the SwissProt database, and filtered statistically significant hits present in Homo sapiens^53^. Details of *in silico* analysis are presented in Supplementary Methods.

### Ethical approval

The research design was approved by the Institutional Review Board of Poznan University of Medical Sciences, Poland. Informed consent was obtained from all study participants. The study was carried out in accordance with the approved guidelines.

## Additional Information

**How to cite this article**: Grzegorzewska, A. E. *et al*. Associations of the calcium-sensing receptor gene *CASR* rs7652589 SNP with nephrolithiasis and secondary hyperparathyroidism in haemodialysis patients. *Sci. Rep*. **6**, 35188; doi: 10.1038/srep35188 (2016).

## Supplementary Material

Supplementary Information

## Figures and Tables

**Table 1:** Demographic, clinical, and biochemical characteristics of haemodialysis (HD) patients.

Parameter	All HD patients n = 1162	HD patients at the initiation of treatment with cinacalcet n = 162
Demographic		
Age at the last data actualization, years	66.1, 17.1–95.9	59.1, 17.1–83.0
Gender, males	506 (56.4)	87 (53.7)
Clinical		
Main causes of end-stage renal disease		
Diabetic nephropathy	335 (28.8)	22 (13.6)
Hypertensive nephropathy	215 (18.5)	27 (16.7)
Chronic glomerulonephritis	170 (14.6)	47 (29.0)
Nephrolithiasis related chronic kidney disease	108 (9.3)	19 (11.7)
Age at RRT onset, years	60.9, 11.1–91.1	51.3, 11.1–77.5
Renal replacement therapy vintage, years	4.69, 0.07–29.5	6.89, 0.07–28.3
Patients with coronary artery disease	416 (35.8)	46 (28.4)
Patients with myocardial infarction	224 (19.3)	32 (19.7)
Parathyroidectomized subjects	33 (2.8)	0 (0.0)
Patients treated with cinacalcet	162 (13.9)	162 (100.0)
Serum^a^		
Total calcium, mg/dl	8.85, 6.01–12.8	9.02 (8.80–11.7)
Phosphorus, mg/dl	5.03, 1.75–12.0	5.40 (2.58–12.0)
Total alkaline phosphatase, U/l	95.8, 13.5–1684	120 (41–1353)
Intact parathyroid hormone, pg/ml	377, 7.33–3757	999 (518–3280)
25-hydroxyvitamin D, ng/ml	13.8, 4.51–30.1^b^	13.7, 5.80–23.4^c^

Results are presented as median and range (maximum–minimum) or number (percentage).

Conversion factors to SI units are as follows: for calcium, 0.2495; 25-hydroxyvitamin D, 2.496; parathyroid hormone, 0.1061; and phosphorus, 0.3229.

^a^Normal serum ranges: total calcium, 8.80–10.20 mg/dl; phosphorus, 2.70–4.50 mg/dl; total alkaline phosphatase, 35–105 U/l; intact parathyroid hormone, 15.0–65.0 pg/ml; 25-hydroxyvitamin D, 30–80 ng/ml.

^b^n = 217.

^c^n = 32.

**Table 2:** *CASR* rs7652589 genotype and allele frequencies in HD patients with and without nephrolithiasis-related ESRD.

*CASR* rs7652589	HD patients with nephrolithiasis-related ESRD	HD patients without nephrolithiasis-related ESRD	Odds ratio (95% CI)	P value	P_*trend*_	P_*genotype*_
	(n, frequency)	(n, frequency)				
	n = 108	n = 1054				
GG	29 (0.27)	396 (0.38)	Reference	—	0.007	0.02
AG	55 (0.51)	509 (0.48)	1.476 (0.923–2.358)	0.1		
AA	24 (0.22)	149 (0.14)	2.199 (1.240–3.900)	0.01		
AA+AG vs GG	79 (0.73)	658 (0.62)	1.639 (1.052–2.555)	0.036^a^		
AA vs AG+GG	84 (0.78)	905 (0.86)	1.735 (1.068–2.821)	0.035^a^		
MAF	(0.48)	(0.38)	1.469 (1.109–1.947)	0.008		
P for HWE	0.830	0.476				

Abbreviations: ESRD, end stage renal kidney disease; HD, haemodialysis; HWE, Hardy-Weinberg equilibrium; MAF, minor allele frequency.

^a^Not significant after the Bonferroni correction.

**Table 3:** *CASR* rs7652589 genotype and allele frequencies in HD patients with serum PTH >500 pg/mL and remaining HD patients.

*CASR* rs7652589	HD patients with serum PTH >500 pg/ml	HD patients serum PTH ≤ 500 pg/ml	Odds ratio (95% CI)	P value	P_*trend*_	P_*genotype*_
	(n, frequency)	(n, frequency)				
	n = 442	n = 720				
GG	153 (0.35)	272 (0.38)	Reference	—	0.035	0.035
AG	208 (0.47)	356 (0.49)	1.039 (0.800–1.350)	0.8		
AA	81 (0.18)	92 (0.13)	1.565 (1.094–2.240)	0.018^a^		
AA+AG vs GG	289 (0.65)	448 (0.62)	1.147 (0.896–1.468)	0.3		
AA vs AG+GG	361 (0.82)	628 (0.87)	1.532 (1.106–2.121)	0.01		
MAF	(0.42)	(0.38)	1.200 (1.011–1.424)	0.041		
P for HWE	0.486	0.141				

Abbreviations: HD, haemodialysis; HWE, Hardy-Weinberg equilibrium; MAF, minor allele frequency; PTH, parathyroid hormone.

^a^Not significant after the Bonferroni correction.

**Table 4:** Results of the transcription factor binding site prediction by software FIMO.

Allele	Matched sequence	Transcription factor	Modification (in presence of the A allele)	Strand	P value	Q value
G	ttgc**G**tgttct	GLI3	Removed	+	6.59E-05	0.0152
G	attgc**G**tgt	AHR	Removed	+	8.53E-05	0.0216
G	gc**G**tgttcttgcagg	TP53	Removed	+	0.000376	0.0435
A	CATGCAATT	PO2F1_f1	−	−	0.000449	0.119
A	gcaacagttcaattgc**A**tg	SOX18	Added	+	0.000101	0.0232
A	ttgc**A**tgttcttgcagggag	TP63	Added	+	0.000214	0.0448
