# Supplementary material for: Associations of the calcium-sensing receptor gene CASR rs7652589 SNP with nephrolithiasis and secondary hyperparathyroidism in haemodialysis patients
Source: Sci Rep. 2016 Oct 14;6:35188. doi: 10.1038/srep35188 (PMC5064403; doi:10.1038/srep35188)
Supplement: Supplementary Information [file srep35188-s1.doc]

**Associations of the calcium-sensing receptor gene *CASR* rs7652589 SNP with nephrolithiasis and secondary hyperparathyroidism in haemodialysis patients**

**Supplementary material**

Alicja E. Grzegorzewska^1*^, Mateusz Paciorkowski^2^, Adrianna Mostowska^3^, Bartosz Frycz^3^, Wojciech Warchoł^4^, Ireneusz Stolarek^5^, Marek Figlerowicz^5^, Paweł P. Jagodziński^3^

^1^ Department of Nephrology, Transplantology and Internal Diseases, Poznan University of Medical Sciences, Poznań, Poland

^2^ Department of Internal Diseases, Pleszew Medical Center, Pleszew, Poland

^3^ Department of Biochemistry and Molecular Biology, Poznan University of Medical Sciences, Poznań, Poland

^4^ Department of Biophysics, Poznan University of Medical Sciences, Poznań, Poland

^5^ Institute of Bioorganic Chemistry, Polish Academy of Sciences, Poznań, Poland

The corresponding author: Prof. Alicja E. Grzegorzewska, FERA. Department of Nephrology, Transplantology and Internal Diseases, Poznań University of Medical Sciences, 49 Przybyszewskiego Blvd., 60-355 Poznań, Poland. Fax: 48 61 8691 688; Mobile phone: 48 696 08 44 87. E-mail: [alicja_grzegorzewska@yahoo.com](mailto:alicja_grzegorzewska@yahoo.com)

***Supplementary Table 1*.** Power analysis for comparison of distribution of *CASR* rs7652589 polymorphism between haemodialysis patients (n = 1162) and controls (n = 918)

|  |  |  | POWER (%) | | |
| --- | --- | --- | --- | --- | --- |
| Population | Allele frequency | Genetic effect^a^ | Genetic model | | |
| risk |  |  | Dominant | Recessive | Log additive |
| 0.0012 | 0.38 | 0.50 | 99.99 | 99.81 | 99.99 |
|  |  | 0.75 | 89.41 | 58.58 | 99.22 |
|  |  | 1.00 | 5.00 | 5.00 | 5.00 |
|  |  | 1.25 | 99.13 | 93.12 | 93.87 |
|  |  | 1.50 | 99.99 | 99.82 | 99.99 |
|  |  | 1.75 | 99.99 | 99.99 | 99.99 |
|  |  | 2.00 | 99.99 | 99.99 | 99.99 |
|  |  | 2.25 | 99.99 | 99.99 | 99.99 |
|  |  | 2.50 | 99.99 | 99.99 | 99.99 |
|  |  | 2.75 | 99.99 | 99.99 | 99.99 |
|  |  | 3.00 | 99.99 | 99.99 | 99.99 |

^a^Odds ratio.

The allelic frequencies of the *CASR* rs7652589 SNP did not deviate from Hardy-Weinberg equilibrium in either the main groups (all HD patients, controls) or in the studied subgroups.

***Supplementary Table 2*.** *CASR* rs7652589 genotype and allele frequencies in haemodialysis patients and healthy controls

| *CASR* rs7652589 | HD patients | Controls | Odds ratio (95% CI) | P value | P*_trend_* | P*_genotype_* |
| --- | --- | --- | --- | --- | --- | --- |
|  | (n, frequency) | (n, frequency) |  |  |  |  |
|  | n = 1162 | n = 918 |  |  |  |  |
| GG | 425 (0.37) | 351 (0.38) | Reference | - | 0.5 | 0.7 |
| AG | 564 (0.49) | 434 (0.47) | 1.073 (0.889-1.296) | 0.5 |  |  |
| AA | 173 (0.15) | 133 (0.14) | 1.074 (0.823-1.403) | 0.6 |  |  |
| AA+AG vs GG | 737 (0.63) | 567 (0.62) | 1.074 (0.898-1.284) | 0.5 |  |  |
| AA vs AG+GG | 989 (0.85) | 785 (0.86) | 1.032 (0.808-1.319) | 0.8 |  |  |
| MAF | (0.39) | (0.38) | 1.044 (0.921-1.184) | 0.5 |  |  |
| P for HWE | 0.525 | 0.951 |  |  |  |  |

Abbreviations: HWE, Hardy-Weinberg equilibrium; MAF, minor allele frequency

***Supplementary Table 3*.** Associations between *CASR* rs7652589 polymorphic variants and selected haemodialysis patient data

|  |  |  |  |  |  |  |
| --- | --- | --- | --- | --- | --- | --- |
| Parameter | GG | AG | AA | Mode |  |  |
| Clinical data, n = 1162 | n = 425 | n = 564 | n = 173 | of inheritance | Odds ratio (95% CI) | P value |
| Age at the renal therapy replacement onset, years | 62.1 | 60.6 | 59.2 | AA vs AG + GG |  | 0.5^a^ |
|  | (11.1 - 90.8) | (13.7 - 91.1) | (14.4 - 85.4) | AA + AG vs GG |  | 0.5^a^ |
|  |  |  |  | AA vs GG |  | 0.0002^a^ |
| Nephrolithiasis-related chronic kidney disease,  n = 108 | 29 (6.8) | 55 (9.8) | 24 (13.9) | AA vs AG + GG | 1.735 (1.068-2.821) | 0.035^b^ |
|  |  |  |  | AA + AG vs GG | 1.639 (1.052-2.555) | 0.036^b^ |
|  |  |  |  | AA vs GG | 2.199 (1.240-3.900) | 0.010^b^ |
| Patients with coronary artery disease, n = 416 | 159 (37.4) | 196 (34.8) | 61 (35.2) | AA vs AG + GG | 0.973 (0.694-1.364) | 0.9^b^ |
|  |  |  |  | AA + AG vs GG | 0.896 (0.699-1.148) | 0.4^b^ |
|  |  |  |  | AA vs GG | 0.911 (0.630-1.317) | 0.7^b^ |
| Patients with a history of myocardial infarction, | 78 (18.4) | 109 (19.3) | 37 (21.4) | AA vs AG + GG | 1.167 (0.785-1.735) | 0.5^b^ |
| n = 224 |  |  |  | AA + AG vs GG | 1.099 (0.810-1.491) | 0.6^b^ |
|  |  |  |  | AA vs GG | 1.210 (0.781-1.877) | 0.5^b^ |
| Parathyroidectomized patients, n = 33 | 11 (2.6) | 18 (3.2) | 4 (2.3) | AA vs AG + GG | 0.784 (0.272-2.258) | 0.8^b^ |
|  |  |  |  | AA + AG vs GG | 1.158 (0.556-2.413) | 0.8^b^ |
|  |  |  |  | AA vs GG | 0.891 (0.280-2.838) | 0.8^b^ |
| Patients treated with cinacalcet, n = 162 | 65 (15.3) | 69 (12.2) | 28 (16.2) | AA vs AG + GG | 1.232 (0.790-1.920) | 0.4^b^ |
|  |  |  |  | AA + AG vs GG | 0.839 (0.598-1.179) | 0.4^b^ |
|  |  |  |  | AA vs GG | 1.069 (0.660-1.734) | 0.9^b^ |
| Patients treated with phosphate binders, n = 1026 | 380 (89.4) | 500 (88.7) | 146 (84.4) | AA vs AG + GG | 0.67 (0.424-1.057) | 0.1^b^ |
|  |  |  |  | AA + AG vs GG | 0.841 (0.575-1.228) | 0.4^b^ |
|  |  |  |  | AA vs GG | 0.640 (0.383-1.071) | 0.1^b^ |
| Laboratory data, n = 1162 |  |  |  |  |  |  |
| Total calcium, mg/dl | 8.83 | 8.81 | 9.05 | AA vs AG + GG |  | 0.7^a^ |
|  | (6.65 - 12.8) | (6.01 - 12.3) | (6.80 - 10.7) | AA + AG vs GG |  | 0.033^a,c^ |
|  |  |  |  | AA vs GG |  | 0.06^a^ |
| Total calcium^d^, mg/dl | 8.40 | 8.40 | 8.87 | AA vs AG + GG |  | 0.0000006^a^ |
|  | (5.12 – 12.8) | (4.64 – 12.1) | (6.56 – 10.7) | AA + AG vs GG |  | 0.2^a^ |
|  |  |  |  | AA vs GG |  | 0.000008^a^ |
| Phosphorus, mg/dl | 5.06 | 5.02 | 4.93 | AA vs AG + GG |  | 0.5^a^ |
|  | (1.75 - 12.0) | (2.03 - 11.0) | (1.95 - 9.33) | AA + AG vs GG |  | 0.2^a^ |
|  |  |  |  | AA vs GG |  | 0.2^a^ |
| Phosphorus^e^, mg/dl | 5.90 | 5.80 | 5.70 | AA vs AG + GG |  | 0.3^a^ |
|  | (1.75 – 16.0) | (2.03 – 13.2) | (1.95 – 11.1) | AA + AG vs GG |  | 0.3^a^ |
|  |  |  |  | AA vs GG |  | 0.2^a^ |
| Total alkaline phosphatase, U/l | 96 | 96 | 94 | AA vs AG + GG |  | 0.6^a^ |
|  | (13.5 - 1684) | (24 - 1299) | (43 - 1353) | AA + AG vs GG |  | 0.6^a^ |
|  |  |  |  | AA vs GG |  | 0.8^a^ |
| Parathyroid hormone, pg/ml | 338 | 376 | 481 | AA vs AG + GG |  | 0.1^a^ |
|  | (12.9 - 3741) | (7.3 - 3757) | (34 - 2992) | AA + AG vs GG |  | 0.099^a^ |
|  |  |  |  | AA vs GG |  | 0.07^a^ |
| Patients with parathyroid hormone ≤ 150 pg/ml, | 82 (19.3) | 92 (16.3) | 29 (16.8) | AA vs AG + GG | 0.943 (0.613-1.452) | 0.9^b^ |
| n = 203 |  |  |  | AA + AG vs GG | 0.822 (0.603-1.120) | 0.2^b^ |
|  |  |  |  | AA vs GG | 0.842 (0.528-1.343) | 0.5^b^ |
| Patients with parathyroid hormone > 500 pg/ml, | 153 (36.0) | 208 (36.9) | 81 (46.8) | AA vs AG + GG | 1.532 (1.106-2.121) | 0.013^b^ |
| n = 442 |  |  |  | AA + AG vs GG | 1.147 (0.896-1.468) | 0.3^b^ |
|  |  |  |  | AA vs GG | 1.565 (1.094-2.240) | 0.018^b,c^ |
| 25-hydroxyvitamin D, ng/ml | 13.65 | 13.9 | 14.25 | AA vs AG + GG |  | 0.9^a^ |
|  | (4.51-30.1) | (5.7-28.5) | (6.6-24.9) | AA + AG vs GG |  | 0.9^a^ |
|  | n = 84 | n = 95 | n = 38 | AA vs GG |  | 0.9^a^ |

| a Mann Whitney test. |
| --- |
| b Chi - square test with Yates correction.  c Not significant after Bonferroni correction.  d The lowest Ca levels among up to 4 last collected values were used, if patients were receiving Ca-containing drugs.  e The highest P levels among up to 4 last collected values were used, if patients were taking phosphate-binding drugs. |
| Results are presented as median and range (minimum – maximum) or number (percentage).  Conversion factors to SI units are as follows: for calcium, 0.2495; 25-hydroxyvitamin D, 2.496; parathyroid hormone, 0.1061; and phosphorus, 0.3229 |
|  |
|  |

***Supplementary Table 4*. *CASR* rs7652589 genotype and allele frequencies in HD patients with and without nephrolithiasis-related ESRD – adjusted for gender and age at RRT onset**

| *CASR* rs7652589 | HD patients with nephrolithiasis-related ESRD | HD patients without nephrolithiasis-related ESRD | Odds ratio (95% CI) | P value | P*_trend_* | P*_genotype_* |
| --- | --- | --- | --- | --- | --- | --- |
|  | (n, frequency) | (n, frequency) |  |  |  |  |
|  | n = 108 | n = 1054 |  |  |  |  |
| GG | 29 (0.27) | 395 (0.38) | Reference | - | 0.007 | 0.02 |
| AG | 55 (0.51) | 507 (0.48) | 1.483 (0.926-2.367) | 0.1 |  |  |
| AA | 24 (0.22) | 147 (0.14) | 2.257 (1.269-4,017) | 0.008 |  |  |
| AA+AG vs GG | 79 (0.73) | 654 (0.62) | 1.651 (1.060-2.572) | 0.03^a^ |  |  |
| AA vs AG+GG | 84 (0.78) | 902 (0.86) | 1.780 (1.09-2.900) | 0.03^a^ |  |  |
| MAF | (0.48) | (0.38) | 1.483 (1.120-1.966) | 0.007 |  |  |
| P for HWE | 0.830 | 0.476 |  |  |  |  |

Abbreviations: ESRD, end stage renal kidney disease; HD, haemodialysis; HWE, Hardy-Weinberg equilibrium; MAF, minor allele frequency; RRT, renal replacement therapy.

P values below 0.05 are indicated using bold font.

^a^Not significant after the Bonferroni correction.

***Supplementary Table 5*.** Power analysis for comparison of distribution of *CASR* rs7652589 polymorphism between haemodialysis patients with (n = 108) and without (n = 1054) nephrolithiasis-related chronic kidney disease

|  |  |  | POWER (%) | | |
| --- | --- | --- | --- | --- | --- |
| Population | Allele frequency | Genetic effect^a^ | Genetic model | | |
| risk |  |  | Dominant | Recessive | Log additive |
| 0.00004 | 0.38 | 0.50 | 92.70 | 53.29 | 99.16 |
|  |  | 0.75 | 29.08 | 15.48 | 47.72 |
|  |  | 1.00 | 5.00 | 5.00 | 5.00 |
|  |  | 1.25 | 18.38 | 12.89 | 33.65 |
|  |  | 1.50 | 46.74 | 33.70 | 80.28 |
|  |  | 1.75 | 72.01 | 59.05 | 97.31 |
|  |  | 2.00 | 87.14 | 79.34 | 99.78 |
|  |  | 2.25 | 94.48 | 91.27 | 99.99 |
|  |  | 2.50 | 97.69 | 96.81 | 99.99 |
|  |  | 2.75 | 99.03 | 98.96 | 99.99 |
|  |  | 3.00 | 99.58 | 99.69 | 99.99 |
|  |  |  |  |  |  |

^a^Odds ratio.

***Supplementary Table 6***. *CASR* rs7652589 genotype and allele frequencies in hypocalcemic, normocalcemic, and hypercalcemic HD patients (the influence of calcium-containing drugs was not analyzed)

| *CASR* rs7652589 | Hypo-calcemic^a^ HD patients | Normo-calcemic^b^ HD patients | Odds ratio (95% CI) | P value | P*_trend_* | P*_genotype_* |
| --- | --- | --- | --- | --- | --- | --- |
|  | (n, frequency) | (n, frequency) |  |  |  |  |
|  | n = 505 | n = 478 |  |  |  |  |
| GG | 192 (0.38) | 179 (0.37) | Reference | - | 0.1 | 0.03 |
| AG | 258 (0.51) | 220 (0.46) | 1.093 (0.833-1.435) | 0.6 |  |  |
| AA | 55 (0.11) | 79 (0.17) | 0.649 (0.435-0.968) | 0.04 |  |  |
| AA+AG vs GG | 313 (0.62) | 299 (0.63) | 0.976 (0.754-1.263) | 0.9 |  |  |
| AA vs AG+GG | 450 (0.89) | 399 (0.83) | 0.617 (0.427-0.893) | 0.01 |  |  |
| MAF | (0.36) | (0.40) | 0.877 (0.730-1.052) | 0.2 |  |  |
| P for HWE | 0.069 | 0.716 |  |  |  |  |

Abbreviations: HD, haemodialysis; HWE, Hardy-Weinberg equilibrium; MAF, minor allele frequency.

Serum total calcium concentration: a < 8.80 mg/dl, b 8.80 – 10.20 mg/dl

***Supplementary Table 7***. *CASR* rs7652589 genotype and allele frequencies in hypocalcemic, normocalcemic, and hypercalcemic HD patients (the influence of calcium-containing drugs was not analyzed)

| *CASR* rs7652589 | Hyper-calcemic^a^ HD patients | Normo-calcemic^b^ HD patients | Odds ratio (95% CI) | P value | P*_trend_* | P*_genotype_* |
| --- | --- | --- | --- | --- | --- | --- |
|  | (n, frequency) | (n, frequency) |  |  |  |  |
|  | n = 179 | n = 478 |  |  |  |  |
| GG | 54 (0.3) | 179 (0.37) | Reference | - | 0.04 | 0.1 |
| AG | 86 (0.48) | 220 (0.46) | 1.296 (0.874-1.920) | 0.2 |  |  |
| AA | 39 (0.22) | 79 (0.17) | 1.636 (1.003-2.670) | 0.06 |  |  |
| AA+AG vs GG | 125 (0.7) | 299 (0.63) | 1.386 (0.958-2.004) | 0.1 |  |  |
| AA vs AG+GG | 140 (0.78) | 399 (0.83) | 1.407 (0.916-2.161) | 0.1 |  |  |
| MAF | (0.46) | (0.40) | 1.293 (1.012-1.652) | 0.046 |  |  |
| P for HWE | 0.911 | 0.716 |  |  |  |  |

Abbreviations: HD, haemodialysis; HWE, Hardy-Weinberg equilibrium; MAF, minor allele frequency.

Serum total calcium concentration: a > 10.20 mg/dl, b 8.80 – 10.20 mg/dl.

***Supplementary Table 8***. *CASR* rs7652589 genotype and allele frequencies in hypocalcemic, normocalcemic, and hypercalcemic HD patients (the influence of calcium-containing drugs was not analyzed)

| *CASR* rs7652589 | Hyper-calcemic^a^ HD patients | Hypo-calcemic^b^ HD patients | Odds ratio (95% CI) | P value | P*_trend_* | P*_genotype_* |
| --- | --- | --- | --- | --- | --- | --- |
|  | (n, frequency) | (n, frequency) |  |  |  |  |
|  | n = 179 | n = 505 |  |  |  |  |
| GG | 54 (0.30) | 192 (0.38) | Reference | - | 0.001 | 0.001 |
| AG | 86 (0.48) | 258 (0.51) | 1.185 (0.804-1.748) | 0.4 |  |  |
| AA | 39 (0.22) | 55 (0.11) | 2.521 (1.515-4.197) | 0.001 |  |  |
| AA+AG vs GG | 125 (0.7) | 313 (0.62) | 1.420 (0.984-2.048) | 0.07 |  |  |
| AA vs AG+GG | 140 (0.78) | 450 (0.89) | 2.279 (1.450-3.582) | 0.001 |  |  |
| MAF | (0.46) | (0.36) | 1.475 (1.155-1.883) | 0.002 |  |  |
| P for HWE | 0.911 | 0.069 |  |  |  |  |

Abbreviations: HD, haemodialysis; HWE, Hardy-Weinberg equilibrium; MAF, minor allele frequency.

Serum total calcium concentration: a > 10.20 mg/dl, b < 8.80 mg/dl.

***Supplementary Table 9*.** Frequency distribution of polymorphic variants in T helper cell cytokine genes between HD patients with and without nephrolithiasis-related end-stage renal disease

| *IL18*  rs360719 | HD patients with nephrolithiasis-related ESRD | HD patients without nephrolithiasis-related ESRD | Odds ratio (95% CI) | P value | P*_trend_* | P*_genotype_* |
| --- | --- | --- | --- | --- | --- | --- |
|  | (n, frequency) | (n, frequency) |  |  |  |  |
|  | n = 98 | n = 774 |  |  |  |  |
| TT | 58 (0.59) | 410 (0.53) | Reference | - | 0.085 | 0.1 |
| CT | 38 (0.39) | 308 (0.40) | 0.872 (0.565-1.347) | 0.6 |  |  |
| CC | 2 (0.02) | 56 (0.07) | 0.253 (0.060-1.063) | 0.07 |  |  |
| CC+CT vs TT | 40 (0.41) | 364 (0.47) | 0.777 (0.507-1.191) | 0.3 |  |  |
| CC vs CT+TT | 96 (0.98) | 718 (0.93) | 0.267 (0.064-1.113) | 0.08 |  |  |
| MAF | (0.21) | (0.27) | 0.733 (0.511-1.049) | 0.1 |  |  |
| P for HWE | 0.325 | 0.984 |  |  |  |  |

| *CCL2*  rs1024611 | HD patients with nephrolithiasis-related ESRD | HD patients without nephrolithiasis-related ESRD | Odds ratio (95% CI) | P value | P*_trend_* | P*_genotype_* |
| --- | --- | --- | --- | --- | --- | --- |
|  | (n, frequency) | (n, frequency) |  |  |  |  |
|  | n = 95 | n = 735 |  |  |  |  |
| AA | 39 (0.41) | 365 (0.50) | Reference | - | 0.059 | 0.2 |
| AG | 45 (0.47) | 317 (0.43) | 1.329 (0.843-2.093) | 0.3 |  |  |
| GG | 11 (0.12) | 53 (0.07) | 1.942 (0.937-4.025) | 0.1 |  |  |
| GG+AG vs AA | 56 (0.59) | 370 (0.50) | 1.416 (0.918-2.186) | 0.1 |  |  |
| GG vs AG+AA | 84 (0.88) | 682 (0.93) | 1.685 (0.847-3.353) | 0.2 |  |  |
| MAF | (0.35) | (0.29) | 1.348 (0.981-1.854) | 0.08 |  |  |
| P for HWE | 0.734 | 0.167 |  |  |  |  |

| *IL12A* rs568408 | HD patients with nephrolithiasis-related ESRD | HD patients without nephrolithiasis-related ESRD | Odds ratio (95% CI) | P value | P*_trend_* | P*_genotype_* |
| --- | --- | --- | --- | --- | --- | --- |
|  | (n, frequency) | (n, frequency) |  |  |  |  |
|  | n = 95 | n = 749 |  |  |  |  |
| GG | 71 (0.75) | 534 (0.71) | Reference | - | 0.4 | 0.7 |
| AG | 22 (0.23) | 192 (0.26) | 0.862 (0.520-1.429) | 0.7 |  |  |
| AA | 2 (0.02) | 23 (0.03) | 0.654 (0.151-2.834) | 0.8 |  |  |
| AA+AG vs GG | 24 (0.25) | 215 (0.29) | 0.840 (0.515-1.369) | 0.6 |  |  |
| AA vs AG+GG | 93 (0.98) | 726 (0.97) | 0.679 (0.157-2.927) | 0.8 |  |  |
| MAF | (0.14) | (0.16) | 0.839 (0.542-1.299) | 0.5 |  |  |
| P for HWE | 0.982 | 0.534 |  |  |  |  |

| *IL12B* rs3212227 | HD patients with nephrolithiasis-related ESRD | HD patients without nephrolithiasis-related ESRD | Odds ratio (95% CI) | P value | P*_trend_* | P*_genotype_* |
| --- | --- | --- | --- | --- | --- | --- |
|  | (n, frequency) | (n, frequency) |  |  |  |  |
|  | n = 96 | n = 770 |  |  |  |  |
| AA | 56 (0.58) | 464 (0.6) | Reference | - | 0.8 | 0.9 |
| AC | 37 (0.39) | 277 (0.36) | 1.107 (0.712-1.721) | 0.7 |  |  |
| CC | 3 (0.03) | 29 (0.04) | 0.857 (0.253-2.906) | 0.8 |  |  |
| CC+AC vs AA | 40 (0.42) | 306 (0.4) | 1.083 (0.704-1.666) | 0.8 |  |  |
| CC vs AC+AA | 93 (0.97) | 741 (0.96) | 0.824 (0.246-2.760) | 0.98 |  |  |
| MAF | (0.22) | (0.22) | 1.038 (0.724-1.488) | 0.9 |  |  |
| P for HWE | 0.567 | 0.290 |  |  |  |  |

| *IL4R* rs1805015 | HD patients with nephrolithiasis-related ESRD | HD patients without nephrolithiasis-related ESRD | | Odds ratio (95% CI) | | P value | P*_trend_* | P*_genotype_* |
| --- | --- | --- | --- | --- | --- | --- | --- | --- |
|  | (n, frequency) | (n, frequency) | |  |  |  |  |  |
|  | n = 97 | n = 886 | |  |  |  |  |  |
| TT | 74 (0.76) | 608 (0.69) | | Reference | | - | 0.1 | 0.3 |
| CT | 21 (0.22) | 242 (0.27) | | 0.713 (0.429-1.184) | | 0.2 |  |  |
| CC | 2 (0.02) | 36 (0.04) | | 0.457 (0.108-1.935) | | 0.4 |  |  |
| CC+CT vs TT | 23 (0.24) | 278 (0.31) | | 0.680 (0.417-1.109) | | 0.3 |  |  |
| CC vs CT+TT | 95 (0.98) | 850 (0.96) | | 0.497 (0.118-2.098) | | 0.5 |  |  |
| MAF | (0.13) | (0.18) | | 0.687 (0.444-1.064) | | 0.1 |  |  |
| P for HWE | 0.940 | 0.169 | |  | |  |  |  |
|  |  |  | |  | |  |  |  |
| *IL13* rs20541 | HD patients with nephrolithiasis-related ESRD | | HD patients without nephrolithiasis-related ESRD | | Odds ratio (95% CI) | P value | P*_trend_* | P*_genotype_* |
|  | (n, frequency) | | (n, frequency) | |  |  |  |  |
|  | n = 97 | | n = 886 | |  |  |  |  |
| CC | 59 (0.61) | | 511 (0.58) | | Reference | - | 0.8 | 0.6 |
| CT | 31 (0.32) | | 326 (0.37) | | 0.824 (0.522-1.300) | 0.5 |  |  |
| TT | 7 (0.07) | | 49 (0.06) | | 1.237 (0.536-2.857) | 0.8 |  |  |
| TT+CT vs CC | 38 (0.39) | | 375 (0.42) | | 0.878 (0.572-1.348) | 0.6 |  |  |
| TT vs CT+CC | 90 (0.93) | | 837 (0.94) | | 1.329 (0.584-3.021) | 0.7 |  |  |
| MAF | (0.23) | | (0.24) | | 0.960 (0.676-1.364) | 0.9 |  |  |
| P for HWE | 0.597 | | 0.950 | |  |  |  |  |

| *IL28B* rs8099917 | HD patients with nephrolithiasis-related ESRD | HD patients without nephrolithiasis-related ESRD | Odds ratio (95% CI) | P value | P*_trend_* | P*_genotype_* |
| --- | --- | --- | --- | --- | --- | --- |
|  | (n, frequency) | (n, frequency) |  |  |  |  |
|  | n = 107 | n = 1033 |  |  |  |  |
| TT | 63 (0.59) | 669 (0.65) | Reference | - | 0.3 | 0.5 |
| GT | 41 (0.38) | 334 (0.32) | 1.304 (0.861-1.973) | 0.3 |  |  |
| GG | 3 (0.03) | 30 (0.03) | 1.062 (0.315-3.579) | 0.9 |  |  |
| GG+GT vs TT | 44 (0.41) | 364 (0.35) | 1.284 (0.856-1.926) | 0.3 |  |  |
| GG vs GT+TT | 104 (0.97) | 1003 (0.97) | 0.964 (0.289-3.215) | 0.95 |  |  |
| MAF | (0.22) | (0.19) | 1.194 (0.849-1.681) | 0.4 |  |  |
| P for HWE | 0.476 | 0.312 |  |  |  |  |

| *IL28B* rs12979860 | HD patients with nephrolithiasis-related ESRD | HD patients without nephrolithiasis-related ESRD | Odds ratio (95% CI) | P value | P*_trend_* | P*_genotype_* |
| --- | --- | --- | --- | --- | --- | --- |
|  | (n, frequency) | (n, frequency) |  |  |  |  |
|  | n = 107 | n = 1033 |  |  |  |  |
| CC | 39 (0.36) | 443 (0.43) | Reference | - | 0.05 | 0.1 |
| CT | 49 (0.46) | 475 (0.46) | 1.172 (0.755-1.820) | 0.6 |  |  |
| TT | 19 (0.18) | 115 (0.11) | 1.877 (1.045-3.371) | 0.05 |  |  |
| TT+CT vs CC | 68 (0.64) | 590 (0.57) | 1.309 (0.867-1.978) | 0.2 |  |  |
| TT vs CT+CC | 88 (0.82) | 918 (0.89) | 1.724 (1.012-2.935) | 0.06 |  |  |
| MAF | (0.41) | (0.34) | 1.322 (0.992-1.763) | 0.07 |  |  |
| P for HWE | 0.870 | 0.765 |  |  |  |  |

Abbreviations: HD, haemodialysis; HWE, Hardy-Weinberg equilibrium; MAF, minor allele frequency.

***Supplementary Table 10.*** An analysis of interaction between *CASR* rs7652589 and polymorphic variants in T helper cell cytokine genes in HD patients with and without nephrolithiasis-related end-stage renal disease

| CHR1 | GENE1 | SNP1 | CHR2 | GENE2 | SNP2 | OR_INT | STAT | P |
| --- | --- | --- | --- | --- | --- | --- | --- | --- |
| 3 | *CASR* | rs7652589 | 3 | *IL12A* | rs568408 | 1.2044 | 0.3485 | 0.5549 |
| 3 | *CASR* | rs7652589 | 5 | *IL13* | rs20541 | 1.1383 | 0.2383 | 0.6254 |
| 3 | *CASR* | rs7652589 | 5 | *IL12B* | rs3212227 | 1.5939 | 2.6150 | 0.1059 |
| 3 | *CASR* | rs7652589 | 11 | *IL18* | rs360719 | 0.7507 | 1.1975 | 0.2738 |
| 3 | *CASR* | rs7652589 | 16 | *IL4R* | rs1805015 | 1.0374 | 0.0137 | 0.9068 |
| 3 | *CASR* | rs7652589 | 17 | *CCL2* | rs1024611 | 0.5652 | 5.193 | 0.0227 |
| 3 | *CASR* | rs7652589 | 19 | *IL28B* | rs12979860 | 0.9821 | 0.0070 | 0.9330 |
| 3 | *CASR* | rs7652589 | 19 | *IL28B* | rs8099917 | 0.8807 | 0.2491 | 0.6177 |

Abbreviations: CHR1, chromosome of first SNP; CHR2, chromosome of second SNP; GENE1, gene of the first SNP; GENE2, gene of the second SNP; OR_INT, odds ratio for interaction; *P*, asymptotic p-value; SNP1, identifier for first SNP; SNP2, identifier for second SNP; STAT, Chi-square statistic, 1df

P values below 0.05 are indicated using bold font.

***Supplementary Table 11*.** Frequency distribution of polymorphic variants in vitamin D pathway genes between HD patients with and without nephrolithiasis-related end-stage renal disease

| *GC* rs7041 | HD patients with nephrolithiasis-related ESRD | HD patients without nephrolithiasis-related ESRD | | Odds ratio (95% CI) | P value | P*_trend_* | P*_genotype_* |
| --- | --- | --- | --- | --- | --- | --- | --- |
|  | (n, frequency) | (n, frequency) | |  |  |  |  |
|  | n = 105 | n = 1036 | |  |  |  |  |
| GG | 31 (0.3) | 351 (0.34) | | Reference | - | 0.3 | 0.5 |
| GT | 52 (0.5) | 504 (0.49) | | 1.168 (0.734-1.860) | 0.6 |  |  |
| TT | 22 (0.21) | 181 (0.17) | | 1.376 (0.774-2.446) | 0.3 |  |  |
| TT+GT vs GG | 74 (0.7) | 685 (0.66) | | 1.223 (0.789-1.897) | 0.4 |  |  |
| TT vs GT+GG | 83 (0.79) | 855 (0.83) | | 1.252 (0.762-2.058) | 0.5 |  |  |
| MAF | (0.46) | (0.42) | | 1.173 (0.882-1.560) | 0.3 |  |  |
| P for HWE | 1.000 | 1.000 | |  |  |  |  |
|  |  |  | |  |  |  |  |
| *VDR* rs2228570 | HD patients with nephrolithiasis-related ESRD | | HD patients without nephrolithiasis-related ESRD | Odds ratio (95% CI) | P value | P*_trend_* | P*_genotype_* |
|  | (n, frequency) | | (n, frequency) |  |  |  |  |
|  | n = 103 | | n = 1026 |  |  |  |  |
| CC | 33 (0.32) | | 298 (0.29) | Reference | - | 0.5 | 0.8 |
| CT | 51 (0.5) | | 515 (0.5) | 0.894 (0.564-1.417) | 0.7 |  |  |
| TT | 19 (0.18) | | 213 (0.21) | 0.806 (0.446-1.455) | 0.6 |  |  |
| TT+CT vs CC | 70 (0.68) | | 728 (0.71) | 0.868 (0.562-1.342) | 0.6 |  |  |
| TT vs CT+CC | 84 (0.82) | | 813 (0.79) | 0.863 (0.513-1.453) | 0.7 |  |  |
| MAF | (0.43) | | (0.46) | 0.898 (0.673-1.199) | 0.5 |  |  |
| P for HWE | 0.996 | | 0.942 |  |  |  |  |

| *VDR* rs1544410 | HD patients with nephrolithiasis-related ESRD | HD patients without nephrolithiasis-related ESRD | Odds ratio (95% CI) | P value | P*_trend_* | P*_genotype_* |
| --- | --- | --- | --- | --- | --- | --- |
|  | (n, frequency) | (n, frequency) |  |  |  |  |
|  | n = 105 | n = 1046 |  |  |  |  |
| GG | 36 (0.34) | 387 (0.37) | Reference | - | 0.9 | 0.7 |
| AG | 54 (0.51) | 492 (0.47) | 1.180 (0.758-1.836) | 0.5 |  |  |
| AA | 15 (0.14) | 167 (0.16) | 0.966 (0.515-1.812) | 0.9 |  |  |
| AA+AG vs GG | 69 (0.66) | 659 (0.63) | 1.126 (0.738-1.717) | 0.7 |  |  |
| AA vs AG+GG | 90 (0.86) | 879 (0.84) | 0.877 (0.496-1.553) | 0.8 |  |  |
| MAF | (0.4) | (0.39) | 1.022 (0.765-1.365) | 0.9 |  |  |
| P for HWE | 0.765 | 0.879 |  |  |  |  |

| *RXRA* rs10776909 | HD patients with nephrolithiasis-related ESRD | HD patients without nephrolithiasis-related ESRD | Odds ratio (95% CI) | P value | P*_trend_* | P*_genotype_* |
| --- | --- | --- | --- | --- | --- | --- |
|  | (n, frequency) | (n, frequency) |  |  |  |  |
|  | n = 108 | n = 1052 |  |  |  |  |
| CC | 73 (0.68) | 638 (0.61) | Reference | - | 0.3 | 0.3 |
| CT | 30 (0.28) | 369 (0.35) | 0.711 (0.456-1.108) | 0.2 |  |  |
| TT | 5 (0.05) | 45 (0.04) | 0.971 (0.374-2.524) | 0.95 |  |  |
| TT+CT vs CC | 35 (0.32) | 414 (0.39) | 0.739 (0.485-1.126) | 0.2 |  |  |
| TT vs CT+CC | 103 (0.95) | 1007 (0.96) | 1.086 (0.422-2.798) | 0.9 |  |  |
| MAF | (0.19) | (0.22) | 0.815 (0.569-1.166) | 0.3 |  |  |
| P for HWE | 0.711 | 0.657 |  |  |  |  |

| *RXRA* rs10881578 | HD patients with nephrolithiasis-related ESRD | HD patients without nephrolithiasis-related ESRD | Odds ratio (95% CI) | P value | P*_trend_* | P*_genotype_* |
| --- | --- | --- | --- | --- | --- | --- |
|  | (n, frequency) | (n, frequency) |  |  |  |  |
|  | n = 107 | n = 1051 |  |  |  |  |
| AA | 62 (0.58) | 525 (0.5) | Reference | - | 0.05 | 0.1 |
| AG | 40 (0.37) | 424 (0.4) | 0.799 (0.526-1.213) | 0.3 |  |  |
| GG | 5 (0.05) | 102 (0.1) | 0.415 (0.163-1.058) | 0.09 |  |  |
| GG+AG vs AA | 45 (0.42) | 526 (0.5) | 0.724 (0.484-1.083) | 0.1 |  |  |
| GG vs AG+AA | 102 (0.95) | 949 (0.9) | 0.456 (0.182-1.145) | 0.1 |  |  |
| MAF | (0.23) | (0.30) | 0.716 (0.514-0.996) | 0.06 |  |  |
| P for HWE | 0.902 | 0.484 |  |  |  |  |

| *RXRA* rs749759 | HD patients with nephrolithiasis-related ESRD | HD patients without nephrolithiasis-related ESRD | Odds ratio (95% CI) | P value | P*_trend_* | P*_genotype_* |
| --- | --- | --- | --- | --- | --- | --- |
|  | (n, frequency) | (n, frequency) |  |  |  |  |
|  | n = 107 | n = 1049 |  |  |  |  |
| GG | 59 (0.55) | 561 (0.53) | Reference | - | 0.7 | 0.9 |
| AG | 41 (0.38) | 415 (0.4) | 0.939 (0.618-1.427) | 0.9 |  |  |
| AA | 7 (0.07) | 73 (0.07) | 0.912 (0.401-2.072) | 0.99 |  |  |
| AA+AG vs GG | 48 (0.45) | 488 (0.47) | 0.935 (0.627-1.395) | 0.8 |  |  |
| AA vs AG+GG | 100 (0.93) | 976 (0.93) | 0.936 (0.420-2.088) | 0.9 |  |  |
| MAF | (0.26) | (0.27) | 0.948 (0.687-1.307) | 0.8 |  |  |
| P for HWE | 0.999 | 0.951 |  |  |  |  |

Abbreviations: HD, haemodialysis; HWE, Hardy-Weinberg equilibrium; MAF, minor allele frequency

***Supplementary Table 12.*** An analysis of interaction between *CASR* rs7652589 and polymorphic variants in vitamin D signaling pathway genes in HD patients with and without nephrolithiasis-related end-stage renal disease

| CHR1 | SNP1 | GENE1 | CHR2 | SNP2 | GENE2 | OR_INT | STAT | P |
| --- | --- | --- | --- | --- | --- | --- | --- | --- |
| 3 | rs7652589 | *CASR* | 4 | rs7041 | *GC* | 0.8352 | 0.7029 | 0.4 |
| 3 | rs7652589 | *CASR* | 4 | rs1155563 | *GC* | 0.9134 | 0.1892 | 0.7 |
| 3 | rs7652589 | *CASR* | 4 | rs2298849 | *GC* | 1.0168 | 0.0044 | 0.9 |
| 3 | rs7652589 | *CASR* | 9 | rs10881578 | *RXRA* | 0.8698 | 0.3243 | 0.6 |
| 3 | rs7652589 | *CASR* | 9 | rs10776909 | *RXRA* | 0.9447 | 0.0442 | 0.8 |
| 3 | rs7652589 | *CASR* | 9 | rs749759 | *RXRA* | 0.8337 | 0.5594 | 0.5 |
| 3 | rs7652589 | *CASR* | 12 | rs1544410 | *VDR* | 0.9894 | 0.0026 | 0.96 |
| 3 | rs7652589 | *CASR* | 12 | rs2228570 | *VDR* | 1.5230 | 3.8099 | 0.05 |

Abbreviations: CHR1, chromosome of first SNP; CHR2, chromosome of second SNP; GENE1, gene of the first SNP; GENE2, gene of the second SNP; OR_INT, odds ratio for interaction; *P*, asymptotic p-value; SNP1, identifier for first SNP; SNP2, identifier for second SNP; STAT, Chi-square statistic, 1df

***Supplementary Table 13***. Effects of treatment with cinacalcet in respect with *CASR* rs7652589 polymorphic variants in haemodialysis patients

| Parameter | *CASR* rs7652589 | | | Model of  inheritance | P value |
| --- | --- | --- | --- | --- | --- |
|  | GG | AG | AA |  |  |
|  | n = 65 | n = 69 | n = 28 |  |  |
| Maximum dose of cinacalcet, mg/day | 60 (30 - 150) | 60 (30 - 180) | 60 (30 - 120) | AA vs AG + GG  AA + AG vs GG  AA vs GG | 0.9  0.1  0.6 |
| Parathyroid hormone at the therapy onset, pg/ml | 984 (549 – 2,588) | 1,017 (518 – 3,280) | 932 (532 – 2,992) | AA vs AG + GG  AA + AG vs GG  AA vs GG | 0.09 0.9  0.2 |
| Parathyroid hormone at 1-2 months after the application of the maximum dose, pg/ml | 440 (27 – 1,867) | 498 (122 – 3,109) | 462 (185 – 2,495) | AA vs AG + GG  AA + AG vs GG  AA vs GG | 0.99 0.3  0.7 |
| Percentual decrease of the initial parathyroid hormone concentration | 53 (-29 - 97) | 48 (-113 - 93) | 48 (-31 - 70) | AA vs AG + GG  AA + AG vs GG  AA vs GG | 0.2 0.08  0.08 |
| Response to cinacalcet, n, % of cases | 51 (78.5) | 43 (62.3) | 21 (75.0) | AA vs AG + GG  AA + AG vs GG  AA vs GG | 0.8  0.1  0.9 |

Results are presented as median and range (minimum – maximum) or number (percentage).

# *Supplementary Figure 1.* The effect of *CASR* rs7652589 genotypes on all-cause mortality in the additive model of inheritance

P = 0.1

# *Supplementary Figure 2.* The effect of *CASR* rs7652589 genotypes on all-cause mortality in the dominant model of inheritance

P = 0.5

# *Supplementary Figure 3.* The effect of *CASR* rs7652589 genotypes on all-cause mortality in the recessive model of inheritance

P = 0.1

***Supplementary Methods***

**Rs7652589 with flanking sequences**

Direct sequences containing one of the rs7652589 alleles were selected from GenBank contig NT-005612.16. The following are the sequences used with FIMO. rs7652589 is in the 102 nucleotide sequence position

>seq1_A_allele

CTGCTTTCATAGAACAGTTTGCTGTTTACTCATATAACCTCCAGTGGTATACTGAGTTGATCACGATCCTCACTCCTTCAGCCTGCAACAGTTCAATTGCATGTTCTTGCAGGGAGGATGATCGTGGAGGCTTCCATTTTGCCATTTGCTCCTCCTCCCCAGAAGTCAAGTTCGTGATTAAATTTTGATTATTTATATTTT

>seq2_G_allele

CTGCTTTCATAGAACAGTTTGCTGTTTACTCATATAACCTCCAGTGGTATACTGAGTTGATCACGATCCTCACTCCTTCAGCCTGCAACAGTTCAATTGCGTGTTCTTGCAGGGAGGATGATCGTGGAGGCTTCCATTTTGCCATTTGCTCCTCCTCCCCAGAAGTCAAGTTCGTGATTAAATTTTGATTATTTATATTTT

**Background file**

Background file was calculated directly from input FASTA sequences with MEME suit fasta-get-markov script. The following, are the contents of the background file used with FIMO.

# seqs: 2 min: 201 max: 201 avg: 201.0 sum: 402 alph: DNA

# order 0

A 2.923e-01

C 2.077e-01

G 2.077e-01

T 2.923e-01

**Overlapping rs7652589 motifs.**

The following are all predicted DNA-binding sites overlapping rs7652589 position for both tested alleles.

**HOCOMOCOv9**

TF allele strand P value q value matched sequence

NR6A1_do seq1_A_allele + 3.84E-05 0.00956 cagttcaattgcA

GLI3_si seq2_G_allele + 6.59E-05 0.0152 ttgcGtgttct

AHR_si seq2_G_allele + 8.53E-05 0.0216 attgcGtgt

SOX18_f1 seq1_A_allele + 0.000101 0.0232 gcaacagttcaattgcAtg

PRGR_f1 seq1_A_allele - 0.00015 0.0297 AGAACAT

GCR_si seq1_A_allele - 0.000201 0.0528 AGAACAT

NR6A1_do seq2_G_allele + 0.00021 0.0262 cagttcaattgcG

PRGR_f1 seq2_G_allele - 0.000216 0.0297 AGAACAC

PO3F1_f1 seq1_A_allele + 0.000217 0.0542 agttcaattgcAtg

ARNT_f1 seq2_G_allele + 0.000277 0.0714 ttgcGtgt

LHX2_f1 seq1_A_allele - 0.000296 0.0571 AACATGCAATTGA

MYBB_f1 seq1_A_allele + 0.000315 0.0811 tgcAtgttct

GCR_do seq1_A_allele - 0.000449 0.092 AAGAACATGCAATTGAAC

PO3F1_f1 seq2_G_allele + 0.000457 0.057 agttcaattgcGtg

GCR_si seq2_G_allele - 0.000507 0.0666 AGAACAC

TFCP2_f1 seq1_A_allele - 0.000522 0.0733 CCCTGCAAGAACATGC

TEAD3_si seq1_A_allele - 0.000523 0.127 CCCTGCAAGAACATG

NR4A1_f1 seq2_G_allele - 0.000604 0.16 CAAGAACAC

EOMES_f1 seq2_G_allele - 0.000683 0.163 CAAGAACACGCAAT

GLI2_f1 seq2_G_allele + 0.000691 0.16 ttgcGtgttct

TFCP2_f1 seq1_A_allele + 0.000704 0.0733 gcAtgttcttgcaggg

P53_f2 seq1_A_allele + 0.000705 0.146 ttgcAtgttcttgcagggag

GCR_do seq2_G_allele - 0.000841 0.092 AAGAACACGCAATTGAAC

P73_si seq1_A_allele + 0.000885 0.0708 attgcAtgttct

PO2F2_si seq1_A_allele - 0.000972 0.258 CATGCAATT

SOX17_f2 seq2_G_allele - 0.000983 0.226 ACGCAATTGAACTGTTGC

**JASPAR CORE REDUNDANT 2016 version 5.0 ALPHA**

TF JASPAR ID allele strand P value q value matched sequence

MA0106.2 seq1_A_allele + 6.81E-05 0.0158 gcAtgttcttgcagg

MA0861.1 seq1_A_allele - 9.82E-05 0.0108 TCCCTGCAAGAACATGCA

MA0861.1 seq2_G_allele - 0.000103 0.0108 TCCCTGCAAGAACACGCA

MA0866.1 seq2_G_allele - 0.000158 0.0188 CGCAATTGAACTGTT

MA0866.1 seq1_A_allele - 0.000158 0.0188 TGCAATTGAACTGTT

MA0006.1 seq2_G_allele + 0.00018 0.0446 tgcGtg

MA0525.1 seq1_A_allele + 0.000214 0.0448 ttgcAtgttcttgcagggag

MA0867.1 seq1_A_allele + 0.000226 0.0136 caacagttcaattgcA

MA0898.1 seq2_G_allele - 0.000242 0.0388 ACACGCAATTGAACTGT

MA0867.1 seq2_G_allele + 0.000247 0.0136 caacagttcaattgcG

MA0627.1 seq1_A_allele - 0.000257 0.0625 GAACATGCAATTGAAC

MA0897.1 seq2_G_allele - 0.000295 0.0418 ACACGCAATTGAACTGT

MA0898.1 seq1_A_allele - 0.000324 0.0388 ACATGCAATTGAACTGT

MA0867.1 seq1_A_allele - 0.000328 0.0136 ATGCAATTGAACTGTT

MA0867.1 seq2_G_allele - 0.000328 0.0136 ACGCAATTGAACTGTT

MA0861.1 seq1_A_allele + 0.000338 0.0236 tgcAtgttcttgcaggga

MA0897.1 seq1_A_allele - 0.00035 0.0418 ACATGCAATTGAACTGT

MA0106.2 seq2_G_allele + 0.000376 0.0435 gcGtgttcttgcagg

MA0160.1 seq2_G_allele - 0.00039 0.108 AAGAACAC

MA0869.1 seq2_G_allele + 0.000424 0.0227 aacagttcaattgcG

MA0014.1 seq2_G_allele + 0.000469 0.0946 tgcGtgttcttgcagggagg

MA0525.2 seq1_A_allele + 0.000478 0.106 tgcAtgttcttgcaggga

MA0869.1 seq1_A_allele + 0.000559 0.0227 ttcaattgcAtgttc

MA0106.3 seq1_A_allele - 0.000565 0.0937 TCCCTGCAAGAACATGCA

MA0640.1 seq2_G_allele - 0.000573 0.135 AACACGCAATTGA

MA0785.1 seq1_A_allele - 0.000582 0.151 AACATGCAATTG

MA0869.1 seq1_A_allele + 0.000586 0.0227 aacagttcaattgcA

MA0866.1 seq2_G_allele + 0.00063 0.0483 aacagttcaattgcG

MA0869.1 seq2_G_allele - 0.000659 0.0227 CGCAATTGAACTGTT

MA0869.1 seq1_A_allele - 0.000659 0.0227 TGCAATTGAACTGTT

MA0787.1 seq1_A_allele - 0.000701 0.182 ACATGCAATTGA

MA0525.1 seq2_G_allele + 0.000701 0.0506 ttgcGtgttcttgcagggag

MA0870.1 seq2_G_allele + 0.000701 0.0915 aacagttcaattgcG

MA0145.2 seq1_A_allele - 0.000719 0.139 CCTGCAAGAACATG

MA0525.1 seq1_A_allele -0.000725 0.0506 CTCCCTGCAAGAACATGCAA

MA0145.1 seq1_A_allele - 0.000727 0.141 CCTGCAAGAACATG

MA0058.2 seq1_A_allele - 0.000734 0.179 AAGAACATGC

MA0870.1 seq1_A_allele + 0.000783 0.0915 aacagttcaattgcA

MA0786.1 seq1_A_allele - 0.0008 0.208 ACATGCAATTGA

MA0137.1 seq2_G_allele - 0.000809 0.057 AGAACACGCAATTG

MA0866.1 seq1_A_allele + 0.000812 0.0483 aacagttcaattgcA

MA0486.2 seq1_A_allele - 0.000847 0.194 TGCAAGAACATGC

MA0507.1 seq1_A_allele + 0.000864 0.221 ttcaattgcAtgt

MA0106.3 seq1_A_allele + 0.000878 0.0937 tgcAtgttcttgcaggga

MA0896.1 seq1_A_allele - 0.000882 0.114 ACATGCAATTGAACTGT

MA0861.1 seq2_G_allele + 0.000885 0.0464 tgcGtgttcttgcaggga

MA0137.1 seq1_A_allele - 0.000895 0.057 AGAACATGCAATTG

MA0663.1 seq2_G_allele + 0.000925 0.236 attgcGtgtt

MA0896.1 seq2_G_allele - 0.000957 0.114 ACACGCAATTGAACTGT

MA0070.1 seq1_A_allele - 0.000975 0.233 AGAACATGCAAT

MA0525.2 seq2_G_allele + 0.000998 0.111 tgcGtgttcttgcaggga
